# The influence of insulin on anticipation and consummatory reward to food intake: A functional imaging study on healthy normal weight and overweight subjects employing intranasal insulin delivery

**DOI:** 10.1002/hbm.26019

**Published:** 2022-07-21

**Authors:** Jed Wingrove, Owen O'Daly, Alfonso De Lara Rubio, Simon Hill, Magda Swedroska, Ben Forbes, Stephanie Amiel, Fernando Zelaya

**Affiliations:** ^1^ Department of Neuroimaging, Institute of Psychiatry Psychology and Neuroscience King's College London London UK; ^2^ Centre for Obesity Research, Department of Medicine University College London London UK; ^3^ Institute of Pharmaceutical Sciences, Pharmaceutical Sciences King's College London London UK; ^4^ Diabetes Research Group, Weston Education Centre King's College London London UK

**Keywords:** anticipation, consummatory, functional imaging, intranasal insulin, overweight

## Abstract

Aberrant responses within homeostatic, hedonic and cognitive systems contribute to poor appetite control in those with an overweight phenotype. The hedonic system incorporates limbic and meso‐limbic regions involved in learning and reward processing, as well as cortical regions involved in motivation, decision making and gustatory processing. Equally important within this complex, multifaceted framework are the cognitive systems involved in inhibitory control and valuation of food choices. Regions within these systems display insulin receptors and pharmacologically increasing central insulin concentrations using intranasal administration (IN‐INS) has been shown to significantly reduce appealing food cue responsiveness and also food intake. In this work we describe a placebo‐controlled crossover pharmacological functional magnetic resonance imaging (fMRI) study that looks at how IN‐INS (160 IU) affects anticipatory and consummatory responses to sweet stimuli and importantly how these responses differ between healthy normal weight and overweight male individuals. This work shows that age matched normal weight and overweight (not obese) individuals respond similarly to both the anticipation and receipt of sweet stimuli under placebo conditions. However, increased central insulin concentrations produce marked differences between groups when anticipating sweet stimuli within the prefrontal cortex and midbrain as well as observed differences in the amygdala during consummatory responses.

## Graphical abstract

This study probes anticipatory and consummatory responses to sweet solution using a fMRI paradigm which delivers liquid stimuli. This work shows that when brain insulin concentration is increased, using intranasal insulin administration, differences in these responses can be identified between lean and overweight individuals.

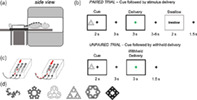

## 1 INTRODUCTION

Research into eating behaviour has provided evidence of three centrally acting mechanisms that interact to modulate energy intake, namely the homeostatic, hedonic and cognitive systems (Berridge, 2009; Berridge & Kringelbach, 2008; Higgs, 2016; Higgs et al., 2017; Higgs & Spetter, 2018; Volkow et al., 2008, 2011). Data suggest that aberrant responses within these systems contribute greatly to the dysregulation of energy intake associated with the overweight phenotype and those living with obesity (Volkow et al., 2011). Obesity is a disease associated with many morbidities, including type 2 diabetes mellitus (T2DM) and increased cardiovascular risk (Nuttall, 2015). Crucial to this work is that even healthy overweight individuals share some risk. Population studies have shown increased T2DM diagnoses (Ganz et al., 2014), cardiovascular related death (Chen et al., 2013) and overall mortality (Bhaskaran et al., 2018) with incremental body mass index (BMI) increases above 25 kg/m^2^, classifying those with an overweight phenotype, but not obese, as an “at‐risk” population for the aforementioned morbidities. In England, approximately 40% of adult males are considered overweight as assessed by BMI, with 33% of individuals classified as normal weight and 26% as obese; summary statistics that classify the majority of males in England as overweight (Barker, 2018).

Of the three systems mentioned the hedonic pathway is characterised by the consumption of palatable food for its potent rewarding effects, as opposed to satisfying the metabolic demands of the body, termed homeostatic. The hedonic system incorporates regions of the limbic system (amygdala, hippocampus, nucleus accumbens [NAcc]) involved in motivation, reinforcement learning and reward processing, as well as cortical regions (prefrontal cortex and insula) involved in motivation, decision making and gustatory processing (Volkow et al., 2011). In addition, the cognitive systems involved in appetite control and top down modulation of inhibitory control and valuation of food choices are important within this complex, multifaceted framework of appetite control (Higgs, 2016).

Insulin is a post‐prandial hormone released by the pancreas following carbohydrate consumption. The primary role of insulin in the body is to regulate and maintain glycaemia through cellular uptake and storage of glucose in skeletal and adipose tissue. In the brain, however, neuronal glucose uptake is insulin independent and has been shown to be centrally active and a large amount of evidence shows that insulin has effects on both homeostatic and hedonic processes (Figlewicz & Benoit, 2009), as well as learning and memory (Kido et al., 2001; Wilcox, 2005). Functional magnetic resonance imaging (fMRI) studies show that acute insulin administration using intranasal application (IN‐INS), significantly reduces appealing food cue responsiveness (Guthoff et al., 2010) and also food intake (Jauch‐Chara et al., 2012). People living with obesity show reduced insulin transport into the cerebrospinal fluid, resulting in decreased concentrations of centrally available insulin and subsequent reductions in central insulin signalling (Craft et al., 1998; Kern et al., 2006). This impaired brain insulin signalling is known as *central insulin resistance*, and is characterised as a physiological state that has been observed alongside peripheral insulin resistance (Heni et al., 2014). It is unknown whether central insulin resistance causes initial weight gain or is a product of obesity (Noakes, 2018). It is known however, that central insulin resistance or a lack of insulin signalling in the brain following a meal leads to alterations in appetite control and changes in food satiation (Schulingkamp et al., 2000). This, therefore, highlights the importance to study individuals prior to the onset of obesity and to see how central insulin resistance affects appetite control mechanisms in individuals who are overweight and not living with obesity. The transport of insulin into the brain decreases with age (Sartorius et al., 2015) and an association between increased dietary fat intake and reduced CNS insulin uptake has been observed in dogs maintained on a high fat diet for 7 weeks (Kaiyala et al., 2000). Nose‐to‐brain delivery of insulin via a nasal pump effectively bypasses the blood brain barrier (BBB) across the olfactory epithelia into the subarachnoid space, reaching brain tissue within 30–60 mins (Banks et al., 2012). IN‐INS can be and has been utilised as a safe research tool for patients or groups where receptor‐mediated transport of insulin across the BBB to the brain tissue may be disrupted (Craft et al., 1998; Kullmann et al., 2015) with limited impact on systemic glycaemia (Schmid et al., 2018). Resting state amplitude of low frequency fluctuation (ALFF) and cerebral blood flow data has shown dose‐dependent effects, with higher doses of IN‐INS (e.g., 160 IU) showing reliable brain effects and have recommended this dose for acute functional imaging studies (Kullmann et al., 2018).

This fMRI investigation seeks to examine the effects of increased central insulin concentrations on hedonic and cognitive brain responsivity to food/liquid stimuli administration in a cohort of normal weight and overweight, but not obese, individuals. This experiment probes the neural basis of the anticipation of food delivery, modelled as the time immediately before receiving a pre‐cued primary food stimulus, as well as the subsequent consummatory response, defined as the response to actual receipt of a primary food stimulus. Anticipation responses to receiving food reward stimuli have been shown to be increased in obese compared to normal weight individuals, known as the hyper‐responsivity theory (Burger & Stice, 2011a, 2011b). In contrast, blood oxygen level dependent (BOLD) effects seen from the consummation response to food stimuli are decreased in obese individuals compared to normal weight, which is considered the hypo‐responsivity theory (Stice et al., 2008). Given these observations, some have hypothesised that these differential effects seen between normal weight and obesity are key features in the development of metabolic disease and dysfunction.

This experiment looks at two discrete responses, anticipation and consummation, for two sweet solutions, one of which contains calories (in the form of sucrose) and the other a non‐nutritive artificial sweetener (“stevia”). The use of non‐nutritive sweeteners as replacements for calorie dense sweeteners such as sucrose has increased over the last decade (Anton et al., 2010). Many suggest non‐nutritive sweeteners can provide a sweet palatable taste while negating the sugar load and post‐prandial spike in insulin associated with many energy dense sweeteners. It has also been proposed that non‐nutritive sweeteners may be useful for promotion of weight loss or weight gain prevention (Mattes & Popkin, 2009), although this remains largely debated, with limited long‐term empirical evidence to support the former.

Stevia, a relatively recent addition to the non‐nutritive sweetener market, is thought to have beneficial effects on insulin sensitivity (Chang et al., 2005) and post‐prandial glucose and insulin levels in comparison to other popular sweeteners and sucrose (Anton et al., 2010). The hedonic effects of stevia, however, have yet to be reported and this study sets out to examine the brain response to this non‐nutritive sweetener. In this study we used Truvia®, which is FDA approved and is one of the leading stevia‐based products on the market in the US with companies such as Coca‐Cola using stevia‐based extracts and sweeteners in some of their products as sugar free alternatives. The main constituents of Truvia® is erythritol, a non‐sugar carbohydrate that belongs to the family of polyols and an extract from the stevia leaf, steviol glycoside.

In this work we describe a crossover pharmacological fMRI study design that looks at how IN‐INS (160 IU) affects anticipatory and consummatory responses to sweet stimuli and importantly how these responses differ between healthy normal weight and overweight, but not obese, male individuals.

## 2 MATERIALS AND METHODS

### 2.1 Participants

This study followed the guidelines of the Declaration of Helsinki and was approved by the King's College London Psychiatry Nursing and Midwifery Ethics Committee (RESCM‐17/18‐2282). Written, informed consent was signed prior to any study procedures. The study comprised three visits, in which visit one was a screening session and the remaining sessions were imaging sessions following one of the two treatments. Imaging sessions were separated by approximately 1 week.

Healthy right‐handed male volunteers were screened to ensure they had no history of psychiatric illness or diabetes diagnosis, no cardiac‐related complications, no history of any eating disorders, asthma or allergies associated with breathing difficulties. During the screening visits, height and weight measurements were taken to ascertain BMI. Only men with a BMI between 18.5 and 30 kg/m^2^ were recruited and stratified into two age‐matched groups defined by BMI as either below (normal weight—*lean*) or above (overweight—*OW*) a BMI of 25 kg/m^2^, for analysis, respectively.

### 2.2 Questionnaires

Refer Supporting Information.

### 2.3 Imaging sessions

For both of the imaging sessions, participants were instructed to follow an overnight (approximately 12 h) fast, with their last meal to be consumed no later than 10 pm the night prior the study visit. Participants abstained from alcohol consumption the night before and caffeine consumption each morning. Shortly after arrival participants provided a blood sample via venepuncture from the cubital vein (referred herein as “pre‐dose”) and a second sample after the MR imaging protocol (referred herein as “post‐scan”), approximately 2.5 h apart. Blood samples were analysed for plasma glucose, serum insulin and serum C‐peptide to assess the effect of IN‐INS administration on peripheral concentrations.

### 2.4 Intranasal administration

Thirty minutes prior to functional image acquisition, participants received either 160 IU insulin (Humulin®, 500 IU/ml, Eli Lily, USA) or saline solution 0.9% w/v (placebo) via intranasal application. Administration was timed so that data acquisition coincided with peak insulin concentrations in the central nervous system, in accordance with the pharmacokinetics of IN‐INS previously reported (Born et al., 2002). The order of treatment administration was counter‐balanced across sessions, and both the subject and researcher were blinded to the treatment (double blind).

Administration was performed using a commercial pump with suitable spray characteristics for nose to brain delivery of insulin solution, using an identical dose to that used in a previous study (Wingrove et al., 2019). Participants had been familiarised with the mechanics of the intranasal pump as well as the application protocol at the screening session and therefore self‐administered the dose under instruction from the lead investigator. Participants took a total of four spray doses of 40 IU in succession, alternating between nostrils and leaving 1 min between each spray to allow time for dissipation and avoid solution running out of the nostrils. Full administration details can be found in Wingrove et al. (2019).

### 2.5 Blood analysis

Baseline measures (pre‐dose) of insulin sensitivity for each participant were calculated using the homeostatic model assessment of insulin resistance (HOMA‐IR) 2 model (Matthews et al., 1985). HOMA‐IR can be calculated using plasma glucose and serum insulin concentrations or plasma glucose with serum C‐peptide concentrations. For this study the latter was implemented using the online, publicly available HOMA‐IR 2 calculator v2.2.3 (https://www.dtu.ox.ac.uk/homacalculator/). Average HOMA‐IR scores across visits were calculated and compared between groups (two sample *t*‐test). The relationship with HOMA‐IR and BMI was explored using a correlation analysis. Pearson's correlation coefficient was calculated to assess this relationship.

The change (Δ) in concentration between pre‐dose and post‐dose collection periods was calculated for each metabolite. These Δ values, for each metabolite, were not normally distributed as assessed using the Anderson Darling normality test (Henderson, 2006). Data were assessed using a non‐parametric rm‐ANOVA, the aligned rank transform (ART) (Wobbrock et al., 2011). Main effects of “Treatment”, “Group” and any interaction effects were interrogated. Significance thresholds for main effects and interaction effects were set to *p* < .05. Significant effects were interrogated with post hoc analysis using the Wilcoxon‐rank test (within‐group or within‐treatment).

### 2.6 Taste delivery paradigm

The taste stimuli used for this paradigm were selected to examine the primary taste response to sweet stimuli. Two sweet stimuli were used; a sucrose solution and a non‐nutritive, low calorie, sweetener solution. In addition, a control mineral water solution was the third taste stimulus. Each taste stimulus was prepared the evening before scanning and was kept at 4°C in the fridge overnight. On the morning of scanning, each taste stimulus was loaded in the designated reservoir prior to subject arrival, approximately an hour before scanning. The taste stimuli maintained a cool temperature by being in the reservoirs. For every visit, this protocol was followed to ensure that the temperature of stimuli was similar across all scanning sessions.

Non‐carbonated bottled water (“Harrogate mineral water”) was used as a control solution for this paradigm. Water is not tasteless and has been shown to activate the taste cortex (Zald & Pardo, 2000). The taste of water however is markedly less intense in comparison to the two sweet stimuli, and therefore was deemed suitable in this instance as a non‐sweet control stimulus.

Sugar or sucrose solution was produced by diluting 12.5 g (approximately three teaspoons) of table sugar (99.99% sucrose) (Caster Baking Sugar, Tate and Lyle) into 150 ml of the water control (bottled Harrogate water—vehicle). This produced a 0.08 g/ml (sucrose/water) stock solution. Each 0.5 ml sucrose bolus delivered contained 0.04 g of sucrose.

Stevia solution was created using a granulated sweetener made from the stevia leaf marketed as Truvia® (The Truvia Company LLC). In reference to the manufacturer's notes, Truvia® is three times sweeter than sugar. Therefore, stevia solution, the name that will be used herein, was produced by diluting 4.2 g (approximately one teaspoon) of Truvia® into 150 ml of bottled water. Each stevia solution stimuli contained 0.014 g of Truvia® from a stock stevia solution of 0.028 g/ml.

The taste outlet mouthpiece was positioned into the right hand‐side of the participant's mouth between the teeth and cheeks. This position ensured comfort for the participant for the duration of this paradigm. The taste outlet positioning was similar for all participants to ensure taste bud stimulation remained similar across all subjects. Participants became familiar with the experimental procedure at the screening session and completed a mock run of the paradigm outside the scanner without the taste stimuli.

**FIGURE 1:**
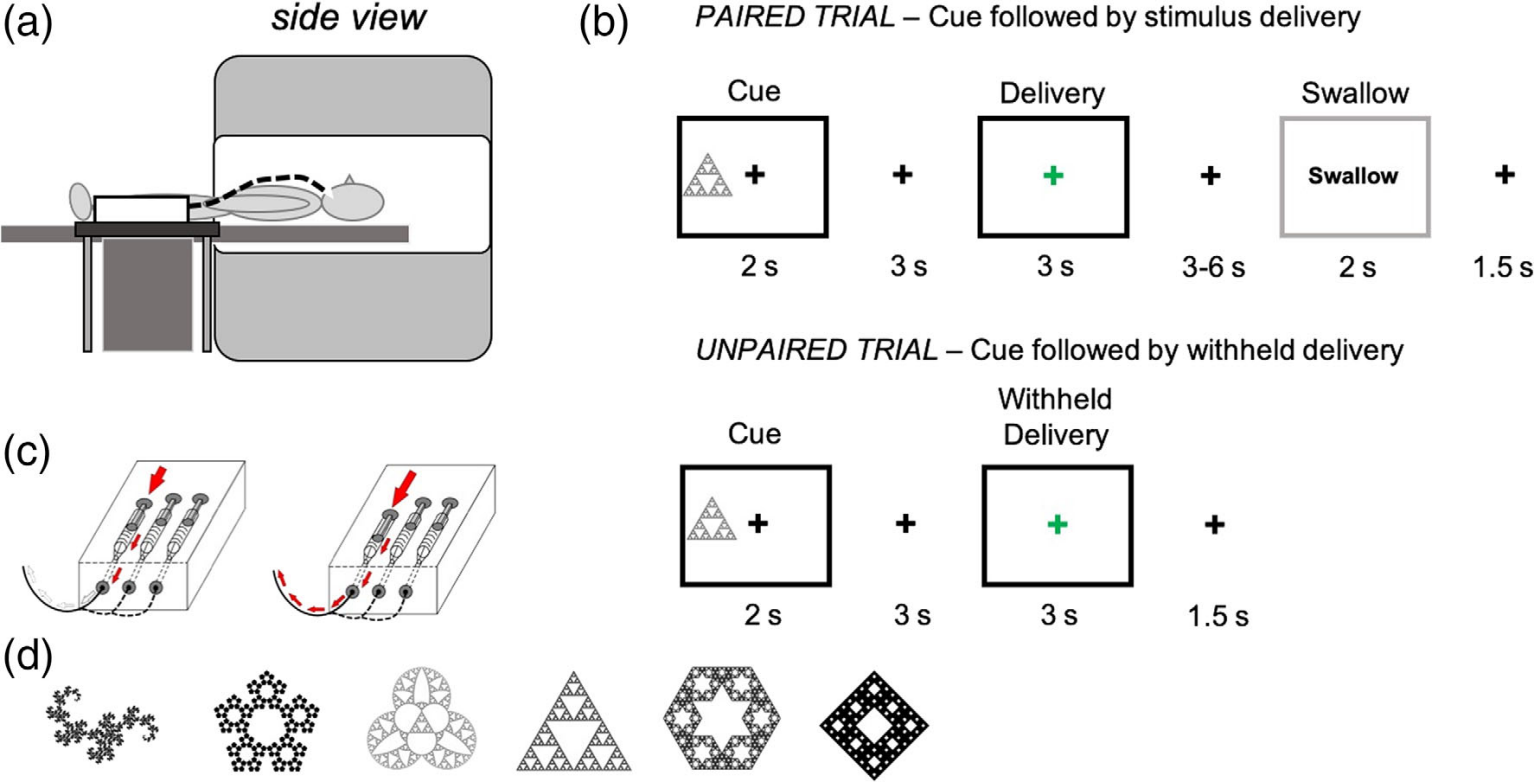
Schematic drawings of the taste dispenser in situ and during bolus delivery. (a) The taste paradigm requires the taste dispenser box to be situated close to the bore of the scanner, either on a side table (seen from this picture) or under the participants legs. The outlet tube and dispenser nozzle are positioned in the participants mouth, inside the scanner. (b) A diagram to show the three different trial types of the taste paradigm. Black boxes represent the events that are modelled as events of interest (cue, delivery and withheld delivery, respectively). Grey boxes represent events that are modelled in the first level model but are not of interest or used in the second level analysis (swallow periods). A paired trial is the most common trial type and involves the delivery of a 0.5 ml bolus/stimulus following visual cue presentation. An unpaired trial does not deliver the 0.5 ml bolus as expected, instead nothing is delivered. (c) Schematic drawings of the syringe pumps that are used to administer the taste bolus, set to 0.5 ml. The two pictures (from left to right) show the evolution of a single taste delivery. (d) The fractal cue images that were used for the taste paradigm

The fluid administration protocol was designed as an “event‐related” paradigm (Figure 1). At the start, one of three abstract, arbitrary fractal images were presented on a screen at the beginning of each trial. Each fractal image was associated with either the water, sucrose, or stevia stimulus and this relationship between presented image and taste stimuli remained constant throughout the entire paradigm for that visit. The cues were not revealed to the participants before the scan, so the participants “learned” which cue belonged to which stimulus as the trial progressed. This cue was presented for 2 s either on the left‐ or the right‐hand side of a white fixation cross, positioned in the middle of the screen. The period between the cue and impending taste stimulus delivery consisted of a white fixation cross and was set to 3 s for every trial. Following this, the cross went from white in colour to green which signalled impending delivery of the taste stimulus. The delivery signal was present for 3 s; however, the taste stimulus (0.5 ml delivery bolus) itself was delivered over the first 1.5 s of this event. Following bolus delivery, the participants were required to keep the liquid bolus in their mouth until the presentation of a “swallow now” prompt. This type of trial is a paired trial, as the cue image is paired with delivery of the taste stimulus. The time spent with the stimulus in the mouth varied from 3 to 6 s and began when the fixation cross returned to white in colour from green. The “swallow now” prompt was presented for 2 s which was followed by a fixed inter trial interval of 1.5 s. Trials were presented in a pseudo‐random order, so that each taste trial was never followed by the same taste. On 40% of the trials the taste stimulus was not delivered, an unpaired trial. This event is termed “withheld delivery”. In these instances, the visual cue (green cross) was presented; however, the stimulus was withheld, referred to as “withheld delivery”. As there was no stimulus delivered, the swallow prompt was not presented/required and the next trial started following the inter‐trial interval. The inclusion of these “withheld delivery” trials permitted the dissociation of the responses related to food anticipation from those associated with the food consumption phase.

An entire run of this paradigm consisted of 45 trials, 9 delivery trials and 6 “withheld delivery” trials for each of the three taste stimuli. For this study 2 runs of this paradigm were performed in succession.

### 2.7 VAS ratings

The first time each taste stimulus was delivered and swallowed two questions followed: “how much do you like the taste?” and “how sweet did you find this taste?”, for which the visual analogue scale (VAS) ranged from 0 for “not at all” to 100 for “a lot or ‘very’”. The participant responded by moving a cursor along a VAS with a button box placed in their right hand. These questions were also presented following the final stimulus, at the end of the second run.

Each attribute (likeness and sweetness) was assessed separately to see if there was any difference in ratings between treatment, groups and stimuli. Likeness ratings were entered into a repeated measures‐analysis of variance (rm‐ANOVA) model with three factors; “Treatment” (PLA, INS), “Group” (LEAN, OW) and “Substance” (water, sugar, stevia) and assessed to *p* < .05. Main effects were interrogated post hoc using Tukey tests, with multiple comparison adjusted *p* values. Similar, to the blood analysis above an ART was used to assess the sweetness ratings as these were not normally distributed. Main effects and interactions were interrogated using the ART model and assessed post hoc using ART contrast tests, with multiple comparisons adjusted *p* values.

### 2.8 Image acquisition

Scanning was conducted using a 3 Tesla MR750 GE Discovery Scanner (General Electric, Waukesha, WI, USA) with a 32‐channel receive only head coil. T1 weighted images were acquired using a 3D Magnetisation Prepared Rapid Acquisition Gradient Recalled Echo (MP‐RAGE) sequence with the following parameters: slice thickness (∆z) = 1.2 mm, slices = 196, TR = 7.312 ms, TE = 3.016 ms, inversion time (TI) = 400 ms, flip angle (FA) = 11°, matrix size (DM) = 256 × 256 with a FOV = 27 cm. Acquisition time: 5:37 min.

Whole brain functional data were acquired using a single‐shot 2D T2* weighted gradient echo echo‐planar imaging (EPI) sequence using parallel imaging (Array coil Spatial Sensitivity Encoding, ASSET). Slices were acquired in a sequential top down direction in the near‐axial plane parallel to the anterior–posterior commissure (AC‐PC) line (approximately 30°) with the following parameters: TR = 2000 ms, TE = 30 ms, flip angle = 75°, matrix size = 64 × 64, FOV = 211 × 211, slice thickness = 3 mm, slice gap = 0.3 mm, no. of slices = 41, in‐plane voxel size = 3.3 × 3.3 mm^2^. Four dummy acquisitions were acquired to achieve steady state magnetisation which were discarded, prior to analysis. The total number of imaging volumes was 324 acquired in 10:56 min for each run. This experiment was conducted, as mentioned earlier, using two consecutive runs.

### 2.9 Image processing

Image processing was performed using a combination of neuroimaging software packages. First, outliers were removed from the time series using 3dDespike [Analysis of Functional Neuroimages (AFNI); Cox, 1996]. To correct for subject motion images were realigned to a base volume using 3dVolreg (AFNI) and all volumes were subsequently corrected for slice timing differences using 3dTshift (AFNI). The base volume was co‐registered to the subject specific T1 anatomical image using epi_reg (FSL, FMRIB, version 3.20, University of Oxford, UK, http://www.fmrib.ox.ac.uk/fsl). Normalisation warping parameters for the anatomical to standard MNI template were then applied using Advanced Normalisation Tools (ANTs) (Avants et al., 2011, 2014). For the final step, spatial filtering of the images was applied using a full width half maximum (FWHM) Gaussian kernel of 8 × 8 × 8 mm using the Statistical Parametric Mapping smoothing function (SPM‐12, Wellcome Trust Centre for Neuroimaging, University College London, UK, http://www.fil.ion.ucl.ac.uk/spm). Furthermore, a high‐pass temporal filter (filter width of 128 s) was also applied as part of the first level model design.

### 2.10 Statistical modelling

For statistical analysis of whole brain data, SPM‐12 software was used. A random effects analysis was implemented through creation of first level contrast images from a standard fixed‐effects general linear model subject‐level analysis. These contrast images were taken through to the second level to interrogate group level effects.

For this paradigm, each trial was modelled as having three conditions of interest; the stimulus cue, stimulus delivery and stimulus delivery withheld. The paradigm was modelled as an event‐related task with condition onsets and durations defined from the task. The duration of the cue presentation was set to 2 s and both the stimulus delivery and withheld conditions were set to 3 s, respectively. Each condition was modelled separately for each of the three taste stimuli, water, sucrose and stevia. In addition, the swallowing periods were implicitly modelled with onsets defined when the swallow cue was presented, 2 s, respectively. The swallow cue was modelled as a single condition as opposed to a taste specific swallow condition. Each condition was convolved with SPM's default canonical haemodynamic response function (Worsley & Friston, 1995). Furthermore, the six “scan‐to‐scan” affine head motion parameters produced during motion correction were included as nuisance regressors in the model to account for subject head motion.

This paradigm was acquired through two functional runs, with identical scanning parameters. Both run 1 and run 2 were combined into a single multisession first‐level analysis. Linear contrasts were generated to examine the differences in BOLD response between each taste for the cue presentation. These linear contrasts were replicated for run 1 and run 2 so that a single contrast statistical parametric map was created for each contrast.

Contrast images were created by comparing the BOLD response to presentation of the cue assigned to one taste against the cue assigned to another. To be clear, the taste cues refer to the fractal images that are presented prior to the taste being delivered, or withheld, depending on trial type. In all cases the cue is 100% predictive of what taste will be delivered, but is not predictive of whether the taste will be delivered or withheld. For this study the focus was on comparing sweet vs. non‐sweet cues and comparing the two types of sweet cues. Therefore, four linear contrast images for each participant were created; sugar cue > water cue, stevia cue > water cue, sugar cue > stevia cue and stevia cue > sugar cue.

Contrast images were generated at the first level to evaluate the BOLD response to stimulus receipt by combining regressors. In the first instance these were water delivery > sweet (sugar + stevia) delivery and sweet delivery > water. We also created an additional two 1st level linear contrast images that focused on the individual taste stimulus by contrasting regressors for taste stimulus delivery with the withheld delivery regressors. These were sugar receipt > sugar receipt withheld and stevia receipt > stevia receipt withheld, referred herein as sugar and stevia receipt responses, respectively. For this study, we also tested the effect of withheld delivery > delivery for both stevia and sugar stimuli. The contrast water delivery > water withheld was not tested in this analysis as the focus was to observe how IN‐INS impacted on sweet stimuli.

#### 2.10.1 Whole brain analysis

Data were analysed for each of the contrasts at the group‐level using SPM‐12. Whole brain contrast images were entered into two random effects, second level, voxel‐wise factorial models (two‐factor ANOVA models) with two factors; “Group” (LEAN, OW) and “Treatment” (IN‐PLA, IN‐INS). The first model, known in SPM as a full factorial repeated measures model, was employed to interrogate the main contrast effects across both groups and IN‐treatments and to also interrogate the main group effect contrasts. Positive and negative contrast *T*‐statistic maps for the main effect of group LEAN versus OW (i.e., LEAN > OW and LEAN < OW).

The second model, known in SPM as a flexible factorial model, includes subject regressors and thereby minimises the contribution of between subject variance to repeated measures analyses (i.e., main effect of treatment and treatment‐by‐group interaction contrasts). For this factorial analysis positive and negative contrast *T*‐statistic maps for the main effect of “treatment” IN‐PLA versus IN‐INS (i.e. either IN‐PLA > IN‐INS or IN‐PLA < IN‐INS), were created as well as interaction effect contrasts “Group” × “Treatment”. This model was not used to interrogate main effects of group as it tends to inflate the significance levels of between‐group comparisons (Gläscher & Gitelman, 2008). This model was used to interrogate treatment and interaction effects. Voxel‐wise whole brain analysis results were made from a cluster‐forming threshold of *p* < .001. In both models, significant clusters were determined based on correction for multiple comparisons computed from “cluster extent” statistics (Friston et al., 1996) using a FWE of *p* < .05. The choice of both the “cluster‐forming” and “cluster extent” thresholds were made to comply with recent recommendations for the use of “cluster‐extent” criteria in fMRI (Woo et al., 2014).

In response to a significant main effect of “Treatment” or Interaction (*p* < .05, FWE) post‐hoc contrasts were tested within each “Group” for the appropriate directionally, using the same cluster‐forming and significance criteria described above.

In response to a significant main effect of “Group” (*p* < .05, FWE) post‐hoc contrasts were tested within each “Treatment” for the appropriate directionally, using the same cluster‐forming and significance criteria described above.

#### 2.10.2 Region of interest analysis

A more focused region of interest (ROI) analysis was employed to compliment the explorative whole brain analysis approach detailed above.

Two sets of anatomical regions were used for this analysis, one set for the cue presentation contrasts and the other set for the taste stimuli contrasts. The bilateral Anterior Cingulate Cortex (ACC), ventro‐medial Prefrontal Cortex (vmPFC) and Nucleus Accumbens (NAcc) were selected a priori to interrogate the cue presentation contrasts. These regions were selected according to previous work which has examined the response to cues associated with conditioned primary rewards (O'Doherty et al., 2002) and have been also linked with insulin‐related reward signals (Tiedemann et al., 2017). The bilateral ACC was designed in SPM‐12 using the Functional MRI tool of the Wake Forest University School of Medicine (http://www.ansir.wfubmc.edu) known as “WFU pick atlas”, implemented with automated anatomical labelling. The Ventromedial PFC was defined from Manning et al. (2015) as two 10 mm radius spheres centred at MNI coordinates, *x* = 6, *y* = 30, *z* = −9 and *x* = −6, y = 24, z = −21. The bilateral NAcc was defined from the Harvard probabilistic brain atlas in FSL (Desikan et al., 2006).

Furthermore, for contrasts relevant for the receipt of the taste stimulus, the anterior insula, amygdala and NAcc regions were interrogated. The bilateral anterior insula was selected as it is heavily involved in taste processing, commonly referred to as the primary gustatory cortex (Frank et al., 2013). The amygdala is a region of the limbic system also involved in response to taste and heavily involved in the gustatory response (Breslin, 2013). And finally, the NAcc, as mentioned, is involved in reward processing and so was used as an ROI for the receipt contrast analysis also. The amygdalae were defined using the WFU pick atlas tool and the anterior insula mask was defined using publicly available cytoarchitecture segmentations (Kurth et al., 2010).

ROI analysis was performed to examine regional effects of “Treatment”, “Group” and also “Treatment” × “Group” interactions. Mean BOLD parameter estimates for each contrast were extracted at the subject level within each ROI mask using 3dmaskave (AFNI). ROI mean BOLD estimates for each contrast were entered into a repeated measures analysis of variance (ANOVA) factorial model with two factors, treatment and group. Significance, for either main effects of treatment or group and also treatment × group interaction effects, was set to *p* < .05 from the rm‐ANOVA. Following the discovery of a significant interaction a post‐hoc Tukey test was used to assess which comparisons were driving the interaction effect. The Tukey post hoc analysis tests every possible comparison and adjusts the *p* value accordingly. For significant main effects of treatment and group, main effects analysis either within group or treatment were used as post hoc tests, respectively. Correction for multiple statistical comparisons was implemented retrospectively using the Benjamin and Hochberg procedure, which controls the false discovery rate (FDR) (Benjamini & Hochberg, 1995). Using this technique, *p* values were adjusted for the number of post hoc tests conducted, and the adjusted *p* values were assessed to a significance threshold of *p* < .05. Only corrected (corr) *p* values will be reported.

Finally, we were interested in determining whether these data may support or align with theories surrounding food intake that relate to the hyper and hypo‐responsiveness of reward related regions in obesity (Davis et al., 2004; Rothemund et al., 2007; Stice et al., 2008). These theories have focused on NAcc activity and dopaminergic transmission in individuals living with obesity and patients with T2DM. To this end we sought to investigate how NAcc BOLD activity from each of the cue presentation contrasts and receipt contrasts correlated with BMI. These analyses were conducted within treatment using Pearson's coefficient. Correction for multiple statistical comparisons was implemented using the Benjamin and Hochberg procedure, which controls the false discovery rate (FDR) (Benjamini & Hochberg, 1995). Using this technique, *p* values were adjusted for the eight correlations conducted, and the adjusted *p* values were assessed to a significance threshold of *p* < .05.

Summary data are presented as mean ± standard deviation (SD) for normally distributed data and using the median and interquartile range (IQR) for non‐normally distributed data, tabulated and in graphical formats where appropriate. All blood, demographic, VAS ratings and ROI statistical analyses were conducted using R statistical analysis software (*Rstudio*—version 1.1453, Boston, MA, http://www.rstudio.org/). Specifically, the “stats” package was used for rm‐ANOVA. Normality tests were performed using the Anderson Darling normality test from the “nortest” package. The non‐normal distributions have been highlighted above (bloods and VAS liking scores), all other measures (extracted ROI data) were normally distributed as tested using the Darling test. Finally, ART models were conducted using the “ARTools” package. Whole brain analysis and first level modelling were conducted using SPM‐12.

## 3 RESULTS

### 3.1 Demographics

Two of the lean participants (as stratified by BMI [kg/m^2^]) were excluded from this analysis due to technical problems encountered with the scanner and taste dispenser apparatus. As a result, the sample size for this analysis has been reduced to 10 Lean (BMI = 22.4 ± 1.9 kg/m^2^, mean ± SD) and 14 overweight individuals (BMI = 27.5 ± 1.7 kg/m^2^). All demographic data are presented in Table 1. These two cohorts were stratified based on BMI and did not differ in age or HOMA‐IR. Furthermore, data on eating behaviour (restraint, inhibition and hunger) did not significantly differ between groups; however, in all cases mean scores were higher than those of the lean group. Finally, both saturated fat intake and sugar intake were comparable between groups (Table S1).

**TABLE 1:** Demographics for the study cohort

	Lean (*n* = 10)	OW (*n* = 14)	*p* value
Age (years)	27.0 ± 5.4	25.0 ± 4.3	.31
BMI (m/kg^2^)	22.4 ± 1.9	27.5 ± 1.7	<.001***
HOMA‐IR	0.87 ± 0.22	1.04 ± 0.37	.17

Abbreviations: Lean, normal weight; OW, overweight; BMI, body mass index; HOMA‐IR, homeostatic model assessment‐insulin resistance. *Notes*: *p* values have been calculated from two sample *t*‐tests. *** Significant *p* < .05. Data are presented as mean ± SD.

### 3.2 Blood analysis

**FIGURE 2:**
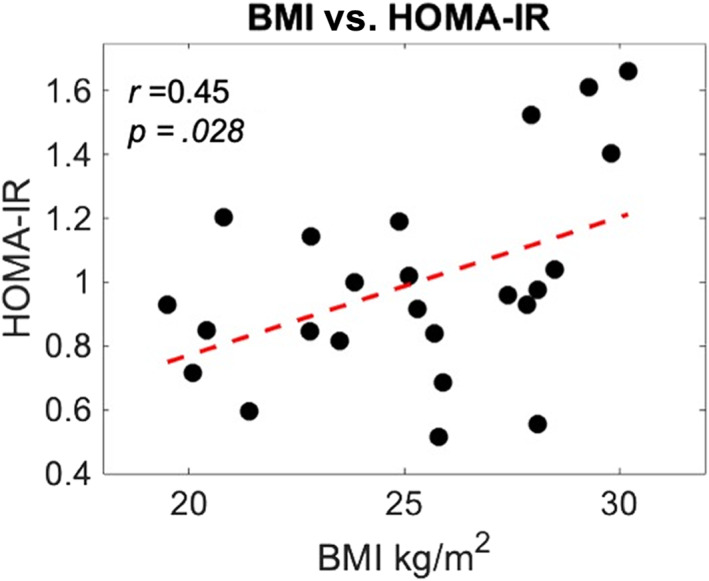
Body mass index (BMI) versus homeostatic model assessment of insulin resistance (HOMA‐IR). Whole group, *n* = 24. Red line = linear coefficient

As mentioned HOMA‐IR values did not significantly differ between groups, although HOMA‐IR was slightly greater in the OW group. Correlation analysis revealed that there was a moderate positive correlation between BMI and HOMA‐IR across both groups (*r* = 0.45, *p* = .027; Figure 2).

Blood metabolite concentration changes (Δ = [post scan] − [pre‐dose]) were interrogated across BMI groups and treatments using a rm‐ANOVA model. Blood analysis was performed on *n* = 23 participants data due to missing samples from one participant. Blood metabolite concentrations were not normally distributed, as assessed using the Anderson Darling normality test. Therefore, a non‐parametric hypothesis test was used; the aligned rank transform (ART; using ARTools package in R) (Wobbrock et al., 2011). Changes in serum insulin did not reveal any significant group, treatment or interaction effects (Table 2). Analysis of Δ plasma glucose and serum C‐peptide concentration revealed a main treatment effect (*F*_(1,42)_ = 5.00, *p* = .031, ART and *F*_(1,42)_ = 6.46, *p* = .014, ART). Post‐hoc tests for plasma glucose did not reveal any within‐group treatment differences (lean: *p* = .15, OW: *p* = .47). Post hoc tests for serum C‐peptide showed a significant treatment effect in the lean group (*p* = .002) but not in the OW group (*p* = .11). The significant effect in the lean group indicated that following IN‐INS the decrease in serum C‐peptide concentration was significantly greater than following IN‐PLA administration (Table 2). *p* values were adjusted for four post‐hoc comparisons.

**TABLE 2:** Tabulated results of the metabolite and hormone analysis

	Lean (*n* = 10)		Overweight (*n* = 13)		*p* values		
	IN‐PLA	IN‐INS	IN‐PLA	IN‐INS	Treatment	Group	Treatment × group interaction
∆ plasma glucose (mmol/l)	−0.05 (0.28)	−0.2 (0.2)	0 (0.1)	−0.1 (0.4)	.031*	.63	.48
∆ serum insulin (mIU/l)	−0.15 (1.18)	−0.75 (13.68)	−1.4 (2,9)	2.0 (8.8)	.41	.33	.61
∆ serum C‐peptide (pmol/l)	−46.5 (64.75)^a^	−103.5 (96.75)^a^	−69.0 (68)	−111 (198)	.015*	.70	.880

*Note*: Analysis was conducted on ∆ (post–pre) levels. A significant treatment effect was seen for serum C‐peptide. Lean (*n* = 10), OW (*n* = 13). * *p* < .05. Data are presented as median (IQR).

^a^ Significant post hoc test, that describes IN‐INS associated C‐peptide suppression over time as greater than IN‐PLA for the lean group only.

### 3.3 VAS assessment

Sweetness ratings did not provide any significant main Treatment (*p* = .51) or Group effects (*p* = .56). A significant Substance effect (*F*_[1,44]_ = 109.36, *p* < .001) was observed. Given there were no Treatment effects post‐hoc Tukey tests were conducted within Treatment.

**FIGURE 3:**
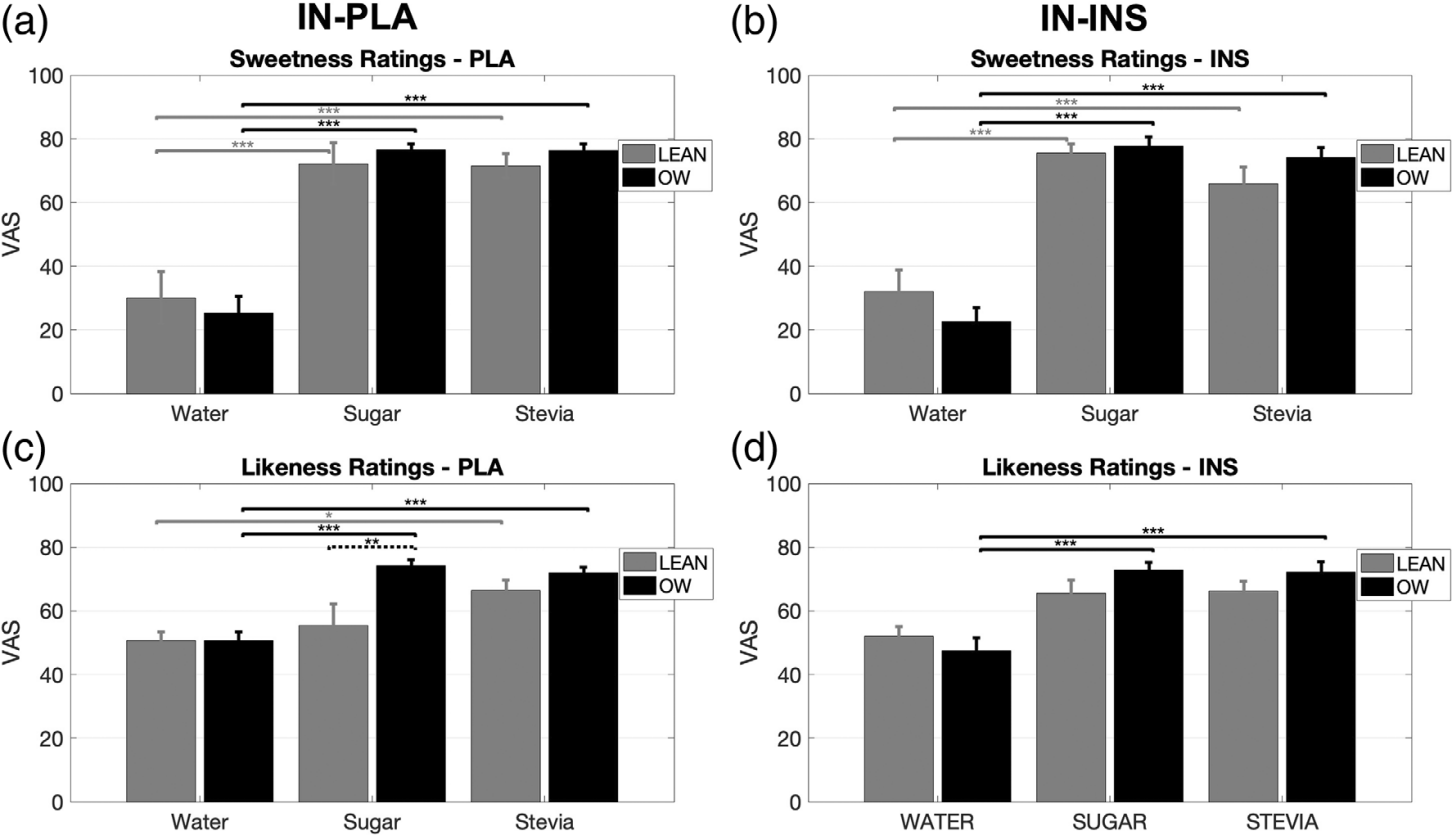
Bar graphs displaying group visual analogue scale (VAS) ratings for sweetness and likeness scores. (a, b) Following both IN‐PLA and IN‐INS, both the lean and OW group found sugar and stevia solution significantly sweeter than water. (c) Following IN‐PLA likeness ratings for sugar solution were significantly higher in the OW group vs. lean. Average ratings in the lean group were significantly greater for stevia over water stimulus, whereas sugar and water were comparable. OW group likeness ratings were significantly greater for both sugar and stevia over water stimulus. (d) Following IN‐INS likeness ratings for water, sugar and stevia were comparable in the lean group. In the OW group, both sugar and stevia likeness ratings were significantly greater than water. Data are presented as mean ± standard error of mean (SEM). Grey bars, lean (*n* = 10). Black bars, overweight (*n* = 14). *p < .05, **p < .01, ***p < .001. Dashed significant bars represent group differences. Solid significant bars represent stimulus differences

Sweetness rating data are presented graphically in Figure 3a,b.

Following IN‐PLA the sweetness rating assessment revealed that the lean group rated water as significantly less sweet than both sugar (*p* < .001) and stevia (*p* < .001). Sugar and stevia sweetness ratings were comparable (*p* = .99). Similarly, in the OW group, water sweetness was significantly lower than both sugar both sugar (*p* < .001) and stevia (*p* < .001). Sugar and stevia sweetness was rated as comparable (*p* = .58) in the OW group. There were no group effects observed between the stimuli following IN‐PLA administration.

Following IN‐INS the sweetness rating assessment revealed that the lean group rated water as significantly less sweet than both sugar (*p* < .001) and stevia (*p* < .001). Sugar and stevia sweetness ratings were comparable (*p* = .41). Similarly, in the OW group, ratings of sweetness for water was significantly lower than both sugar both sugar (*p* < .001) and stevia (*p* < .001). Sugar and stevia sweetness was rated as comparable (*p* = .75) in the OW group. There were no group effects observed for any of the stimuli following IN‐INS administration.

Likeness ratings did not show any significant main Treatment effects (*p* = .7). Significant main Group (*F*_[1,44]_ = 8.04, *p* = .005) and Substance (*F*_[1,44]_ = 43.03, *p* < .001) effects were observed as well as a significant Substance × Group interaction (*F*_[1,44]_ = 5.17, *p* = .007). To interrogate these results and avoid unnecessary multiple comparisons post hoc Tukey tests were conducted within Treatment.

Likeness rating data are presented graphically in Figure 3c,d.

Substance effects within each group are presented first, followed by group effects for each substance. Following IN‐PLA the likeness rating assessment revealed that in the lean group water scores were comparable with sugar (*p* = .91) and stevia (*p* = .063). Additionally, likeness scores for sugar and stevia did not differ in the lean group (*p* = .46). In the OW group, ratings for sugar and stevia were comparable (*p* = .74), whereas water likeness was rated less than sugar (*p* < .001) and stevia (*p* < .001). The only difference between groups was seen for the sugar stimuli (*p* = .004), where liking ratings were lower in the lean group compared to OW subjects.

Following IN‐INS the likeness rating analysis revealed that in the lean group ratings across stimuli did not differ significantly (water vs. sugar: *p* = .17, water vs. stevia: *p* = .12, sugar vs. stevia: *p* = .99). In the OW group, liking ratings for sugar and stevia were comparable (*p* = .99), whereas water likeness was rated less than sugar (*p* < .001) and stevia (*p* < .001). There were no group effects observed for any of the stimuli following IN‐INS administration.

### 3.4 Whole brain fMRI analysis

The water cue > sugar cue and water cue > stevia cue linear contrasts did not provide any significant clusters when looking at the positive effect of this contrast and therefore was not included in the following analysis. Suggesting that the cues associated for both sweet tastes showed greater whole brain BOLD responsiveness compared to water, detailed below.

#### 3.4.1 Sugar cue > water cue

**FIGURE 4:**
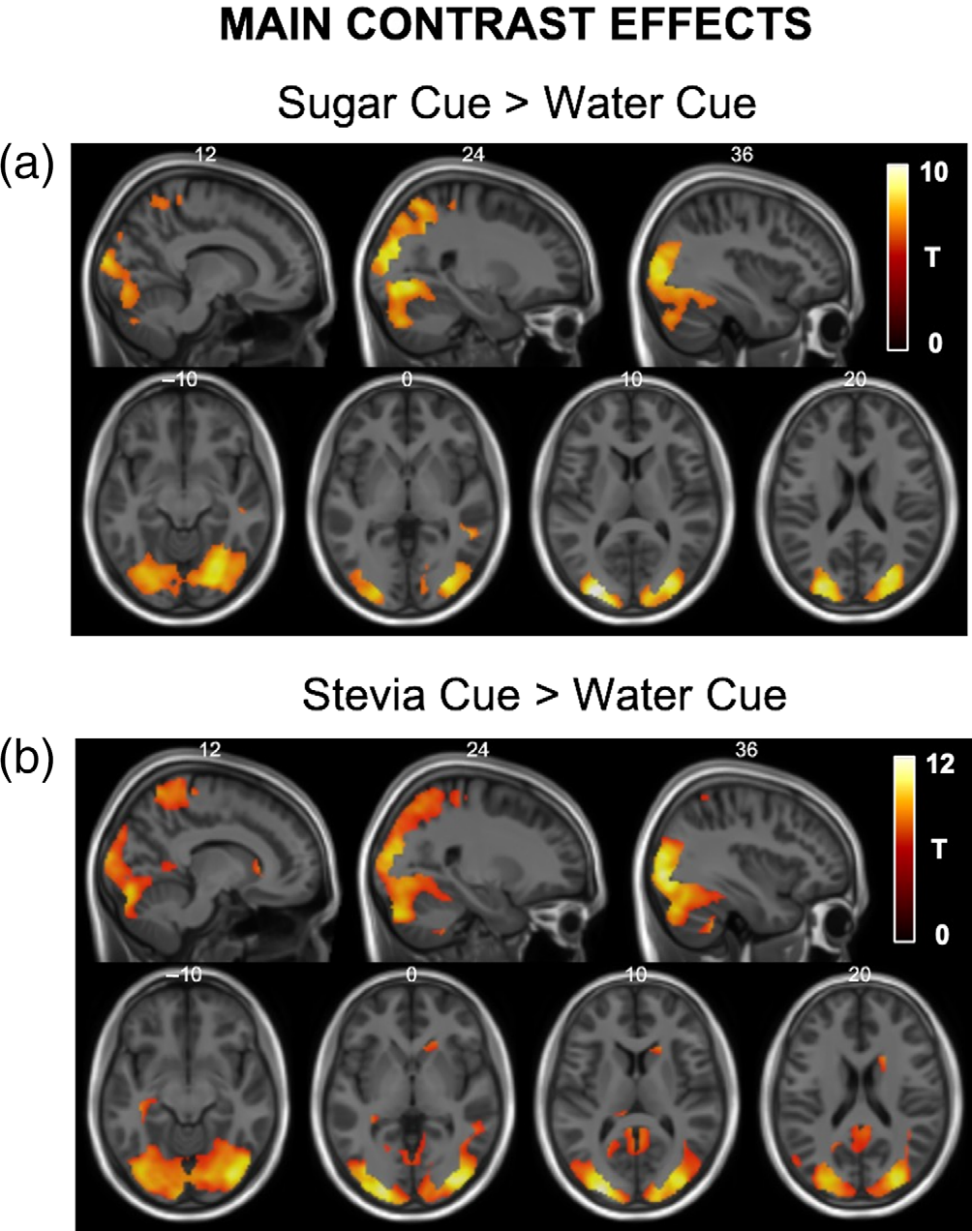
Statistical T‐map for main cue contrast effects overlaid onto structural MNI images. (a) The sugar cue > water cue linear contrast showed statistically significant areas of activation within the bilateral visual association cortex and extrastriate cortex. (b) The stevia cue > water cue linear contrast also showed statistically significant areas of activation within the bilateral visual association cortex, extrastriate cortex and also the right caudate. MNI co‐ordinates are labelled at the top of each slice and only significant clusters are displayed

Whole brain statistical maps for the main contrast effect are shown in Figure 4a. This contrast showed significant BOLD responses in the left and right visual association cortex in addition to the extrastriate cortex (Table 3). Full factorial analysis did not provide any significant group effects in either direction and likewise, the flexible factorial analysis did not provide any significant clusters when interrogating main effects of treatment in either direction nor any interaction effects.

**TABLE 3:** List of significant regions, *p* values, cluster size, *T* scores and peak MNI‐coordinates for the sugar cue > water cue and stevia cue > water cue main contrast effects

Region	*p* value (FWE‐corrected)	Cluster size	*T*‐score	Peak MNI coordinates
Whole brain statistics				
Sugar cue > water cue				
Left and right visual association cortex, extrastriate cortex	<.001	11,964	10.55	−28 −90 10
			8.99	28 −72 −6
			8.78	30 −84 8
Stevia cue > water cue				
Left and right visual association cortex, extrastriate cortex	<.001	22,999	12.10	−28 −94 8
			11.34	34 −86 −2
			10.96	32 −86 16
Boundary of right caudate	<.001	218	7.34	18 2 26

#### 3.4.2 Stevia cue > water cue

Whole brain statistical maps for the main contrast effect are shown in Figure 4b. This contrast showed significant BOLD responses in the left and right visual association cortex, the extrastriate cortex and also along the boundary of the right caudate (Table 3). Full factorial analysis did not provide any significant group effects in either direction and likewise the flexible factorial analysis did not provide any significant clusters when interrogating main effects of treatment in either direction nor any interaction effects.

#### 3.4.3 Sugar cue versus stevia cue

The main contrast effect of this contrast did not provide any significant clusters, suggesting that there is no difference between the BOLD response elicited from presentation of the cue for either sweet stimuli at the whole brain level. Group and treatment effects were therefore not tested.

### 3.5 Water receipt versus sugar and stevia receipt

**FIGURE 5:**
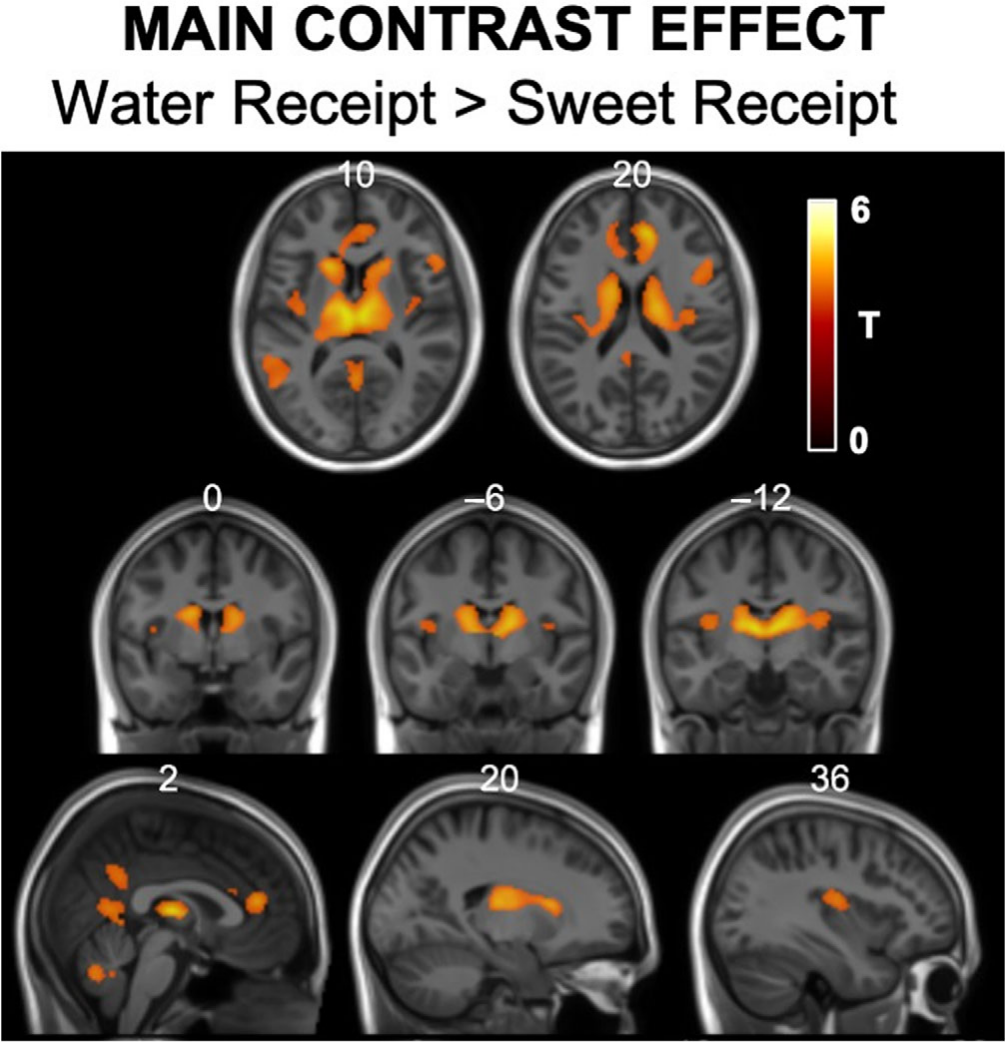
Statistical T‐maps for main the water receipt > sweet (sugar + stevia) receipt main contrast effect overlaid onto structural MNI image. These statistical maps display greater BOLD responsiveness from water in comparison to sweet tasting stimuli in the bilateral dorsal ACC, thalamus, putamen, right operculum, posterior cingulate and cerebellum. MNI co‐ordinates are labelled at the top of each slice and only significant clusters are displayed.

Two contrasts were generated to see how the BOLD response differed between receipt of both sugar and stevia stimuli compared to water (water receipt vs. sugar + stevia receipt). From these two contrasts, we only found significant cluster formations for the water receipt > sweet receipt effect. Whole brain statistical maps for this contrast effect are shown in Figure 5. This contrast produced a significantly greater BOLD response in regions of the left and right thalamus, insula, angular gyrus, cerebellum and dorsal posterior cingulate cortex as well as regions of the cerebellum (Table 4). These differences will be discussed in the discussion section of this article.

**TABLE 4:** List of significant regions, *p* values, cluster size, *T* scores and peak MNI‐coordinates for the water receipt > sugar and stevia receipt main contrast effect

Region	*p* value (FWE‐corrected)	Cluster size	*T*‐score	Peak MNI coordinates
Whole brain statistics				
Water receipt > sugar + stevia receipt				
Left and right prefrontal cortex, thalamus, putamen and caudate	<.001	4081	7.69	8 40 18
Left and right posterior cingulate cortex	<.001	454	5.91	0 −58 10
Right inferior frontal gyrus and operculum	<.001	316	6.15	56 18 14
Left middle temporal cortex	<.001	310	5.96	−54 −46 14
Cerebellum right IX	<.001	231	5.74	8 −54 ‐34

The statistical significance of the main task effects for all the contrasts described above was in fact very high in spite of the relatively small number of subjects scanned; so much so that they survived testing at a much tighter statistical threshold (Voxel‐wise Bonferroni FWE correction *p* < .05). These results are shown at this level of significance in Figures 4a,b a 5 and also Tables 3 and 4.

In addition, delivery of sweet stimuli did not elicit any BOLD responses greater than those seen from water delivery and we did not identify group, treatment or interaction effects from whole brain analysis. This study was designed to address the BOLD effects of taste stimuli with lean and overweight individuals and whether this response is modulated by administration of IN‐INS. To address these aims we therefore focus on the separate contrasts that interrogated the BOLD response to sugar receipt and stevia receipt separately.

#### 3.5.1 Sugar receipt

**FIGURE 6:**
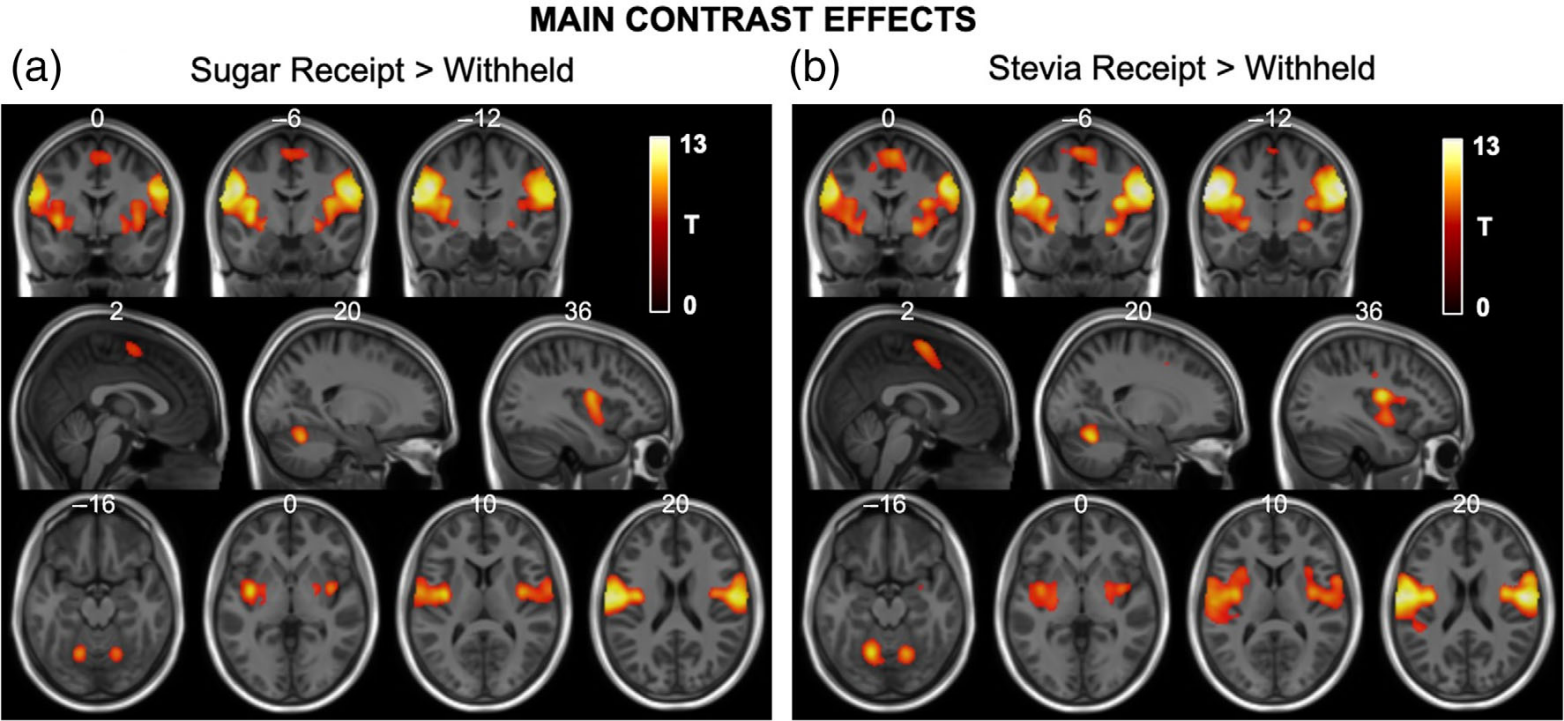
Statistical T‐maps for main receipt contrast effects overlaid onto structural MNI images. (a) The sugar receipt > withheld linear contrast showed statistically significant areas of activation within the bilateral oral somatosensory regions, insula cortex, putamen, supplementary motor area as well as regions of the cerebellum. (b) Similarly, the stevia receipt > withheld linear contrast also showed statistically significant areas of activation within the bilateral oral somatosensory regions, insula cortex, putamen, supplementary motor area as well as regions of the cerebellum. MNI co‐ordinates are labelled at the top of each slice and only significant clusters are displayed

Whole brain statistical maps for this main contrast effect are shown in Figure 6a. This contrast showed significant BOLD responses in the left and right oral regions of the somatosensory cortex, insula cortex, regions of the basal ganglia (putamen and caudate), supplementary motor cortex and the VI of the cerebellum (Table 5). From this contrast no significant whole brain group, treatment or interaction effects in either direction were observed.

**TABLE 5:** List of significant regions, *p* values, cluster size, *T* scores and peak MNI‐coordinates for the sugar receipt > sugar withheld and stevia receipt > stevia withheld main contrast effects

Region	*p* value (FWE‐corrected)	Cluster size	*T*‐score	Peak MNI coordinates
Whole brain statistics				
Sugar receipt > receipt withheld				
Left somatosensory cortex, insula cortex	<.001	4272	13.16	−50 −10 30
Right somatosensory cortex, insula cortex	<.001	3711	12.51	56 −4 26
Left and right supplementary motor cortex	<.001	341	6.22	0 −4 60
Cerebellum left VI	<.001	308	8.67	−18 −62 −20
Cerebellum right VI	<.001	284	8.44	20 −62 −22
Stevia receipt > receipt withheld				
Left somatosensory cortex, insula cortex	<.001	6048	14.74	−50 8 30
Right somatosensory cortex, insula cortex	<.001	4873	14.17	64 −14 28
Left and right supplementary motor cortex	<.001	1282	8.00	6 −2 60
Cerebellum left VI	<.001	707	10.10	−16 −62 −16
Cerebellum right VI	<.001	625	9.88	18 −62 −22

#### 3.5.2 Stevia receipt

**FIGURE 7:**
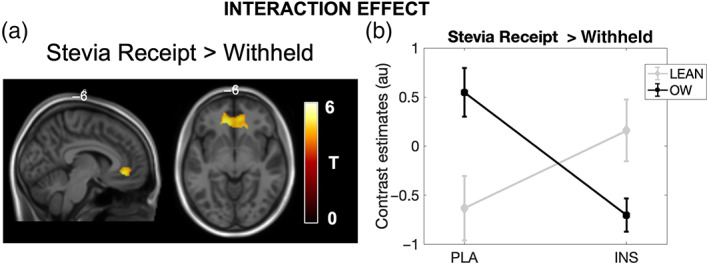
Statistical T‐maps overlaid onto a structural MNI image of the significant treatment × group interaction effect. (a) A significant interaction was observed in the region of the dorsal ACC, (b) plotted are the contrast estimates extracted from this cluster for both lean and OW groups for each treatment, light grey = Lean, black = Overweight (OW). No statistics were performed on the extracted parameter estimates. Lean (*n* = 10), OW (*n* = 14)

Whole brain statistical maps for this main contrast effect are shown in Figure 6b. Similar to sugar receipt described above, this contrast showed significant BOLD responses in the left and right oral regions of the somatosensory cortex, insula cortex, regions of the basal ganglia (putamen and caudate), supplementary motor cortex and the VI of the cerebellum (Table 5). From this contrast no significant group or treatment effects in either direction were observed. When testing for whole brain interaction effects; however, a significant cluster was identified (500 voxels, *p* = .043, *T* = 5.25 [−8, 38, −8] FWE_corr_, Figure 7) in the region of the left dorsal ACC (Brodmann area 32) and left orbitofrontal cortex (Brodmann area 11) which spanned into the right hemisphere also. The interaction described IN‐INS related BOLD responses in the lean group as greater than following IN‐PLA and for the OW group the directionality of treatment effects was the opposite (IN‐PLA > IN‐INS), respectively. These within‐group treatment contrasts were tested as post hoc whole brain analysis. No significant clusters were identified from either whole brain post hoc tests.

Likewise, the statistical significance of the main task effects for the two receipt contrasts described above was in fact very high and a much tighter statistical threshold (Voxel‐wise Bonferroni FWE correction *p* < 0.05) has been applied for these effects. These results are shown at that level of significance in Figure 6a, b and also Table 5.

#### 3.5.3 Withheld > delivery

Withheld delivery phases were contrasted against the delivery phase to identify any regions that encode the effect of not receiving a primary taste stimuli. Whole brain analysis did not find any main contrast effects and so was not carried forward into further flexible factorial or ROI analysis.

### 3.6 ROI analysis

The ROI analysis focused on two cue contrasts, sugar > water and stevia > water, and two sweet stimuli receipt contrasts, sugar receipt and stevia receipt, respectively. ROI analysis was performed to look at group, treatment and interaction effects. Following the same structure as the whole brain analysis these results will be described for each contrast in turn. The relationship between BOLD parameters extracted from the NAcc and BMI, within treatment, was also compared for each contrast.

#### 3.6.1 Sugar > water cue

**FIGURE 8:**
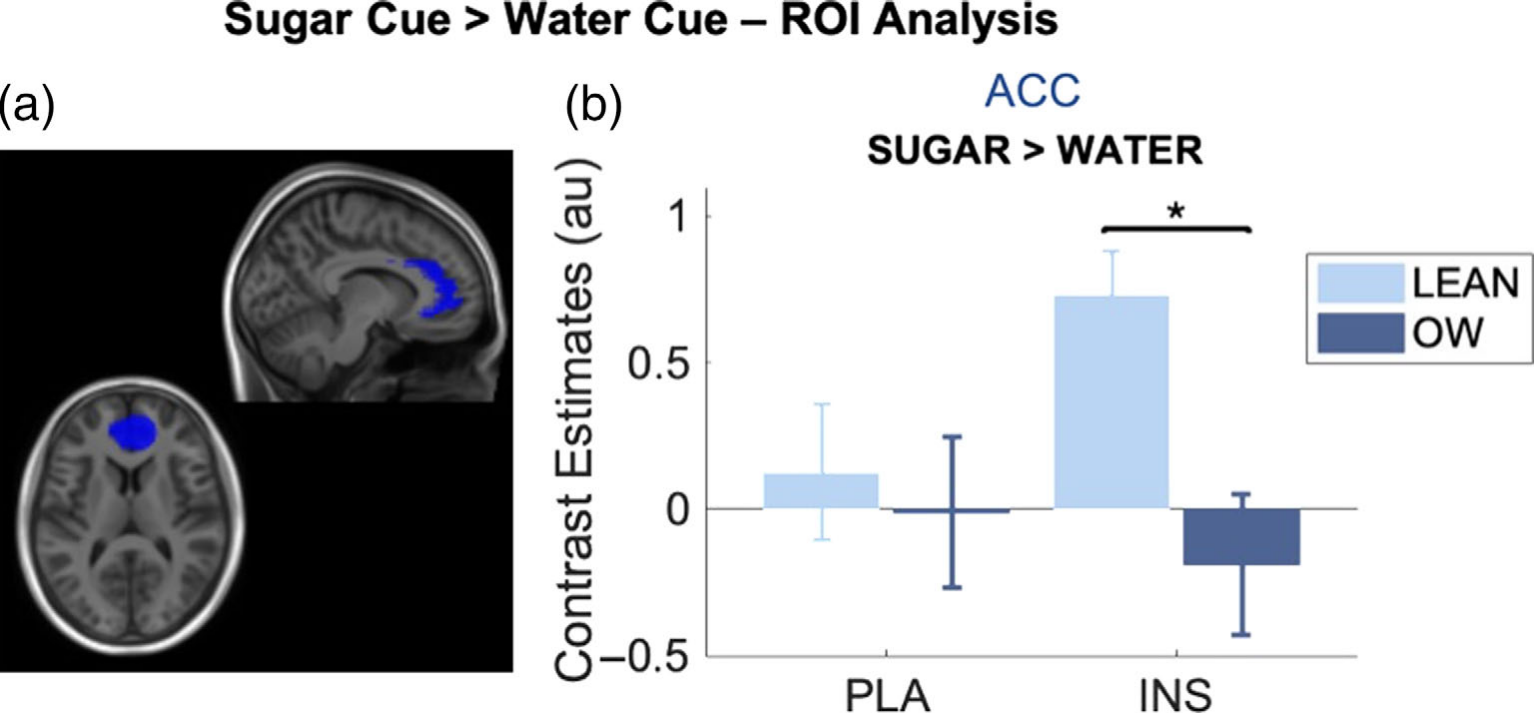
ROI analysis from the (a) ACC ROI (blue), (b) bar graph showing extracted contrast estimates from the ACC in both groups and under both IN‐PLA and IN‐INS conditions for the sugar cue > water cue contrast. Estimates are from contrast images. A significant difference between lean and OW group contrast estimates was observed in this region, exclusively under insulin conditions. Data are presented as mean ± SEM, *p < .05. Lean (*n* = 10), OW (*n* = 14)

ROI analysis for this event revealed a significant group effect within the ACC (*F*_1,44_ = 4.84, *p* = .03). Post hoc tests showed a greater contrast estimate in the lean group compared to the OW group following IN‐INS administration (*F*_1,44_ = 8.55, *p* = .04 _corr_) but not in the placebo session (*F*_1,44_ = 0.14, *p*_corr_ = .71) (presented in Figure 8b). Furthermore, BOLD response results from the vmPFC revealed a significant treatment by group interaction effect (*F*_1,44_ = 5.65, *p* = .022). Tukey test post‐hoc analysis did not, however, produce any significant comparisons, adjusted for multiple comparisons. Analysis of the NAcc did not provide any group (*p* = .86), treatment (*p* = .45) or interaction effects (*p* = .07). Furthermore, correlational analysis of NAcc BOLD response and BMI was not significant for either IN‐PLA (*r* = 0.13, *p*_corr_ = .63) or IN‐INS (*r* = −0.38, *p*_corr_ = .17).

#### 3.6.2 Stevia > water cue

**FIGURE 9:**
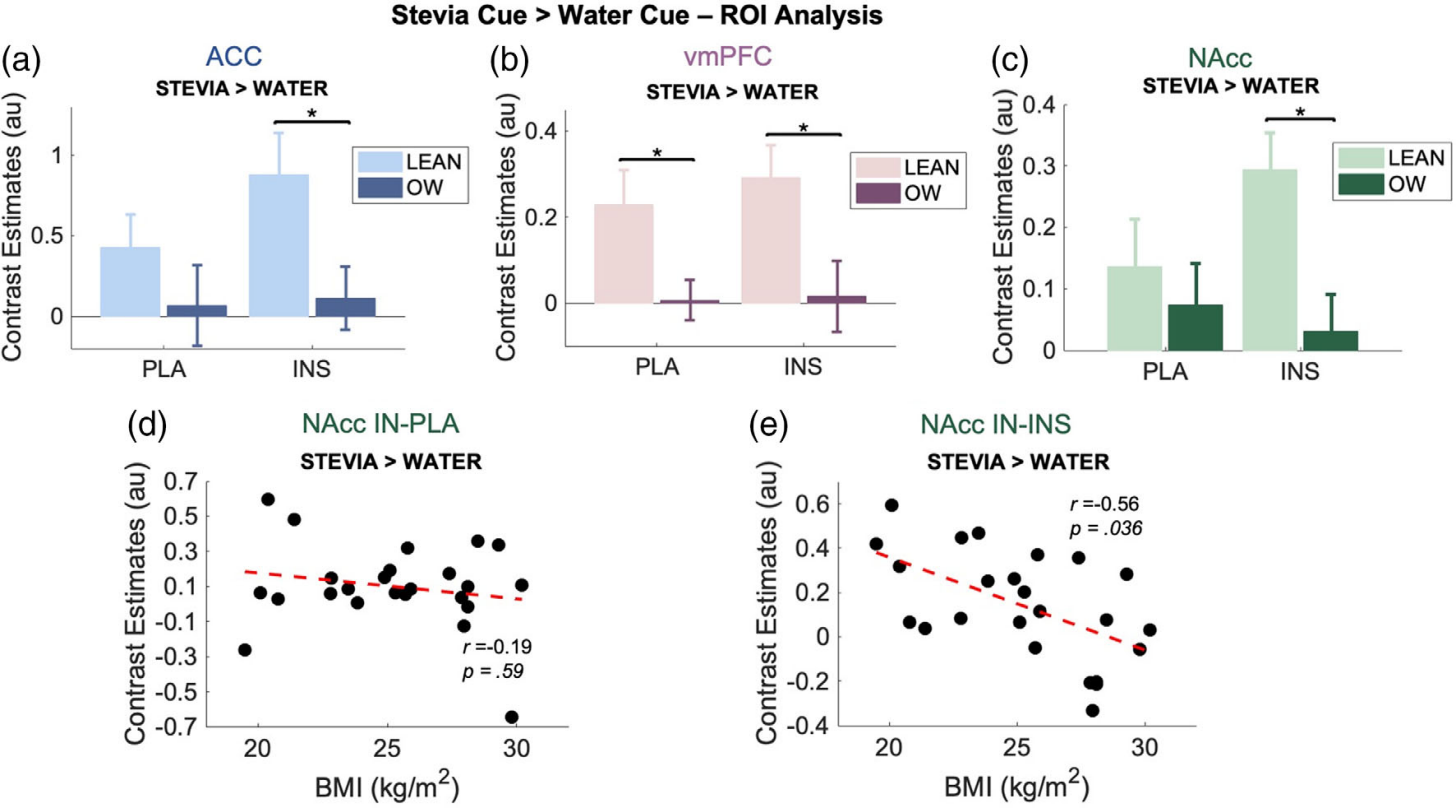
ROI analysis for the stevia cue > water cue contrast. (a–c) bar graph showing extracted contrast estimates from the ACC, vmPFC and NAcc. Estimates are from contrast images. A significant difference between lean and OW group contrast estimates was observed in these regions, for the ACC and NAcc exclusively under insulin conditions, but following both treatments in the vmPFC. Data are presented as mean ± SEM, *p < .05. Lean (*n* = 10), OW (*n* = 14). (d, e) BMI versus NAcc contrast estimates within each treatment. A significant negative relationship was seen following IN‐INS (e) but not IN‐PLA (d). Whole group, *n* = 24. Red line = linear coefficient

ROI analysis for this event revealed a significant group effect within the ACC (*F*_1,44_ = 5.73, *p* = .02). Post hoc tests showed a greater contrast estimate in the lean group compared to the OW group following IN‐INS administration (*F*_1,44_ = 5.78, *p*_corr_ = .045) but not in the placebo session (*F*_1,44_ = 1.08, *p*_corr_ = .39) (presented in Figure 9a). BOLD response results from the vmPFC revealed a significant group effect (*F*_1,44_ = 11.74, *p* = .001). Post‐hoc analysis showed a greater contrast estimate in the lean group compared to the OW group following IN‐PLA administration (*F*_1,44_ = 6.45, *p*_corr_ = .045) and also in the IN‐INS session (*F*_1,44_ = 5.65, *p*_corr_ = .045; presented in Figure 9b). Analysis of the NAcc BOLD estimates also provided a significant group effect (*F*_1,44_ = 5.90, *p* = .02) in the same direction as the ACC and vmPFC (Lean > OW). Post hoc tests showed a greater contrast estimate in the lean group compared to the OW group following IN‐INS administration (*F*_1,44_ = 9.24, *p*_corr_ = .04) but not in the placebo session (*F*_1,44_ = 0.36, *p*_corr_ = .61) (presented in Figure 9c).

Furthermore, correlational analysis of NAcc BOLD response and BMI was not significant under IN‐PLA conditions (*r* = −0.19, *p*_corr_ = .59, Figure 9d) but displayed a moderate but significant negative relationship following IN‐INS administration (*r* = −0.56, *p*_corr_ = .036, Figure 9e).

#### 3.6.3 Sugar receipt

ROI analysis did not reveal any group, treatment or interaction effects for any of the regions tested (anterior insula, amygdala and NAcc). Correlational analysis between NAcc and BMI did however show a moderate positive relationship under IN‐INS conditions (*r* = 0.44) but did not survive multiple comparison correction (*p*_uncorr_ = .029, *p*_corr_ = .16).

#### 3.6.4 Stevia receipt

**FIGURE 10:**
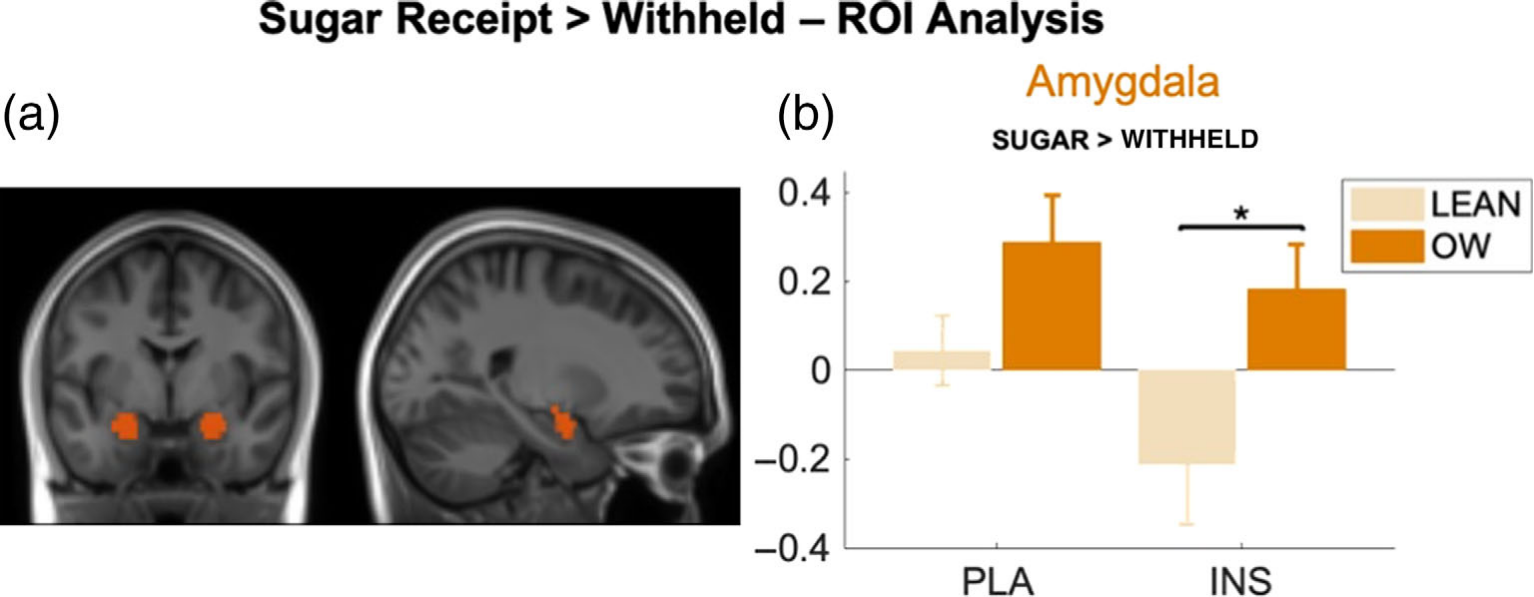
ROI analysis from the (a) amygdala ROI (orange), (b) bar graph showing extracted contrast estimates from the amygdala in both groups and under both IN‐PLA and IN‐INS conditions for the sugar receipt > withheld contrast. Estimates are from contrast images. A significant difference between lean and OW group contrast estimates was observed in this region, exclusively under insulin conditions. Data are presented as mean ± SEM, *p < .05. Lean (*n* = 10), OW (*n* = 14)

ROI analysis revealed a significant group effect for the amygdala BOLD estimates (*F*_1,44_ = 5.73, *p* = .005). This difference described the Lean group BOLD estimates as significantly lower that the OW group. Post hoc testing showed that this difference was significant only following IN‐INS (*F*_1,44_ = 5.64, *p*_corr_ = .045, Figure 10b) but not IN‐PLA (*F*_1,44_ = 2.98, *p*_corr_ = 0.14). No significant effects were observed from either the anterior insula and NAcc and there was no relationship between NAcc estimates and BMI for either treatment.

## 4 DISCUSSION

This study explored the haemodynamic brain changes involved in primary reward processing from conditioned taste stimuli and has shown that IN‐INS administration highlights differences in BOLD responsiveness within regions of the prefrontal cortex, as well as reward and gustatory systems between normal weight and overweight individuals. Despite this study having a pharmacological element the results gathered from this data are largely centred around differences seen as a result of BMI. These groups were comparable across age, peripheral insulin sensitivity (HOMA‐IR), certain eating behaviours (TFEQ) and overall sugar and fat diet composition/intake (DFS), rendering these differences even more relevant when considering brain changes or differences as a consequence of increased body mass. Despite no difference in peripheral insulin sensitivity between these two groups our correlational analysis did show a positive relationship between HOMA‐IR and BMI, further supporting previous HOMA‐IR research that has shown positive correlations or relationships with BMI (Vogeser et al., 2007). The correlation seen from our data was arguably driven by a small group of individuals with both high BMI and high HOMA‐IR scores rather than a uniform linear increase across the group.

Blood was sampled on two occasions during the present protocol. Sampling serial blood measurements throughout the duration of the scanning protocol might have been preferable and is sometimes used for pharmacological fMRI research, as drug activity can be closely correlated with pharmacokinetic data (Mehta & O'Daly, 2011). In this instance, however, temporally rich blood sampling was not feasible, and it was considered more desirable not to disturb the participant during scanning. Regardless, we found that between the two blood sampling time points there was no effect of IN‐INS on glycaemia when looking at groups individually and IN‐INS administration did not produce peripheral insulinemia, which further encourages the use of IN‐INS as a safe pharmacological tool for investigating insulin brain function (Schmid et al., 2018). C‐peptide, a reliable marker of pancreatic insulin release, levels decreased over time under both treatments but in the lean group this decrease was significant following IN‐INS when compared to IN‐PLA. This observation is compatible with either systemic spill‐over of exogenous insulin that, whilst not statistically significant, was able to significantly inhibit endogenous insulin secretion or posits towards a centrally regulated negative feedback pancreatic system that has been suggested previously (Elahi et al., 1982; Schmid et al., 2018).

This is the first time that the primary effects of food (solution) taste and ingestion have been explored as part of a pharmacological investigation with IN‐INS. Previous fMRI reports exploring the associations between elevated insulin and food related processing have largely focused on brain responsiveness to food cues/pictures (Guthoff et al., 2010; Kroemer et al., 2013) as a surrogate marker for food related neural processing. This study, on the other hand, has made use of a primary reward paradigm to explore BOLD effects seen from the anticipation and receipt of sweet stimuli. The paradigm and study were designed to probe the hedonic aspects of appetite control rather than the homeostatic process and therefore the focus of this discussion will be on these aspects, respectively. As mentioned, this study did not provide significant treatment effects of IN‐INS for any of the cue or receipt events tested. We think that this lack of treatment effect could be attributed to acquiring functional data post optimal or maximal insulin concentrations within the central nervous system (CNS). The only published study looking at human pharmacokinetics has shown that IN‐INS (40 IU) peaks at approximately 45 min post administration (Born et al., 2002). Unfortunately, for this study we acquired resting state cerebral blood flow data (Wingrove et al., 2019) to correlate with this peak prior to this paradigm and so this choice may have impacted the treatment effect or lack thereof.

This was an experimental study of reward processing that used a fractal cue to condition individuals prior to a primary gustatory stimulus. The fractal cues had not been seen previously until the morning of each scan and did not contain any patterns or characteristics that were predictive of the taste stimuli. Under IN‐PLA both groups showed that the average BOLD response in primary and secondary visual regions was greater for the two sweet stimuli compared to water. This is in line with previous work where predictive cues coupled with higher value reward stimuli show greater activation within the primary visual extrastriate cortices (Anderson et al., 2014). The increased salience of greater reward‐value cues, once learned, is thought to be augmented by dopaminergic activity, thus priming the brain to seek or find these learned rewards (Hickey et al., 2010). Sweetness scores were greater for sucrose and stevia over water stimuli for both lean and overweight groups and there was no difference between these scores following placebo or insulin or between the two groups. For liking scores, there were no significant effects of treatment. The only group difference was following placebo where sugar was rated higher by the overweight group compared to the lean group, a difference that was not identified following IN‐INS. After looking at the results in more detail two lean individuals found the sugar stimuli slightly too sweet, but only following placebo and not insulin. For the overweight group both sweet stimuli received higher ratings compared to water across treatments. However, in the lean group this trend was not seen across treatments and only under placebo was stevia likeness scores higher than water. The whole brain maps which show increases in BOLD responsiveness in the visual regions for sweet > water cue contrasts do not seem surprising, given how each substance was rated. The extrastriate cortex makes up a part of the secondary visual cortex, receiving inputs directly from the primary visual cortex and is involved with encoding shapes and edges (Okusa et al., 2000; Vinberg & Grill‐Spector, 2008). Presentation of the cue elicited an “anticipatory” brain response, recruiting regions not just involved in visual processing, but those involved in higher level reward processing such as the vmPFC and dorsal ACC, all commonly associated with evaluation and decision making in response to rewarding stimuli (Wallis & Kennerley, 2011).

ROI analysis was used for a more focused analysis of these higher‐level regions. The ACC in particular has been previously shown to be involved in the prediction of rewarding outcomes (Silvetti et al., 2013) and also has been recruited during assessing the probability of receiving primary rewards (Vassena et al., 2014). When looking at the sugar cue > water cue contrast BOLD responses extracted from this region were greater in normal weight versus overweight individuals following IN‐INS only. Similarly, ACC ROI analysis for the stevia cue contrast also revealed significant group differences which followed the same trend; normal weight greater than overweight under IN‐INS conditions only. These results together suggest that the anticipation of a sweet rewarding stimulus, caloric or not, engages this prefrontal evaluative region and that the normal weight phenotype presents a sensitivity to increased central insulin concentrations which is not present in an overweight phenotype.

Looking at the stevia cue contrast (stevia > water), ROI analysis of the vmPFC, showed increased engagement in the normal weight individuals under both conditions. The vmPFC is associated with valuation of stimuli, both food and non‐food related (Tiedemann et al., 2017) as well as decision making. As the task implemented in the current study was passive, in that participants had no choice, the effects seen are hard to decipher and without a decision making element introduced to this paradigm. When anticipating stevia delivery following IN‐PLA NAcc BOLD responsiveness remained inseparable between groups. However, on the IN‐INS study day normal weight BOLD estimates extracted from the NAcc were greater in normal weight compared to overweight, a difference that also displayed a significant negative correlation.

We looked at the linear relationship between NAcc BOLD activity for each of the events with BMI to try and relate our data with the hypo and hyper‐responsive dopaminergic theories of obesity (Burger & Stice, 2011a, 2011b; Volkow et al., 2011). For the stevia cue contrast, the lack of NAcc correlation with BMI under placebo conditions suggests that our data does not support an overweight hyper‐responsivity food cue theory. Rather, in the presence of insulin the NAcc shows increased responsivity with lower BMI. It is unknown whether this trend can be extrapolated to suggest lower BOLD responsivity in those living with obesity. Furthermore, BOLD data are not able to ascertain whether these changes in BOLD reactivity during anticipation are associated with dopaminergic transmission specifically.

Work on reward processing has focused on the concept of reinforcement learning (Rescorla & Wagner, 1972; Schultz, 2001) and the involvement of the dopaminergic system in facilitating and reinforcing reward seeking and approach behaviours. Midbrain dopaminergic projections from the Ventral Tegmental Area and substantia nigra nuclei to the striatum and pre‐frontal cortex are highly implicated in reward processing (Rolls, 2004; Schultz, 2001). Particular focus has been on the NAcc, a region commonly known for its role in pleasure and addiction (Adinoff, 2004). Insulin plays a dual role in the local limbic regions. Firstly, insulin binds to insulin receptors on pre‐synaptic dopamine terminals and acts to increase pre‐synaptic dopamine uptake via upregulated dopamine transporter activity (DAT) (Stouffer et al., 2015). Second, excitatory cholinergic interneurons that synapse onto striatal neurons express insulin receptors. Successful binding of insulin upon these cholinergic interneurons increases excitability, action potential frequency, and acetyl‐choline release and in turn increases downstream dopaminergic release from striatal dopamine neurons that express nicotinic acetylcholine receptors (Stouffer et al., 2015). The latter effect is most prominent upon increased insulin concentrations and is sufficient to overcome the increased DAT uptake activity in response to increased striatal insulin concentrations. The NAcc has the highest density of insulin receptors within the striatum (Werther et al., 1987). The increase in BOLD response seen from the NAcc under IN‐INS from the normal weight group may be explained by the excitatory effects of insulin on cholinergic interneurons associated with subsequent dopamine release. Cholinergic interneurons receive input from the thalamus and this mechanism is involved in directing attention, reinforcement and also learning (Smith et al., 2011). It could be suggested that the insulin‐related BOLD increase in this region is reflective of an informative signal, potentially due to a change in hedonic properties or valence, but nonetheless is a mechanism that we cannot firmly apply to our data at this current moment. Interestingly, Stouffer et al., showed that the sensitivity of insulin related dopamine release from striatal neurons (NAcc, caudate‐putamen) is diet dependent with obese rats displaying diminished insulin related dopamine synaptic concentrations (Stouffer et al., 2015). Although the participants involved in the current study have an overweight and not obese BMI, this result is in support of the notion that insulin may have a differential response in those with an increased BMI.

We must first address our finding that BOLD responses across prefrontal regions as well as the thalamus and basal ganglia following water receipt were greater in amplitude than those of both the sweet stimuli combined. We had intended to use the water as a control taste stimulus. Water is not tasteless and does elicit a taste response (Zald & Pardo, 2000). A factor that could have influenced this response is the variability of the sweet stimulus receipt amongst subjects. This was tested and although the variability was greater, we also found that BOLD responses were in fact lower upon receipt but higher upon cue presentation for sweet stimuli, whereas the opposite was observed for water (lower for cue and higher for receipt). This may suggest a shift of activity from the receipt to the cue phase only in the sweet stimulus and not water and hence the results we have reported. This would be worth investigating in future studies with a larger cohort. We could however compare both sweet stimuli and found that there was no difference in BOLD responsiveness to ingestion of either sugar or stevia‐based solution. It is known that water is not “tasteless” (Zald & Pardo, 2000) and these results show the taste of bottled mineral water elicits a reliable BOLD response irrespective of insulin or placebo challenge. Previous fMRI studies that investigated “taste” often use either a tasteless artificial solution (O'Doherty et al., 2003; Stice et al., 2008) that mimics the ionic complexity of saliva or distilled water (Haase et al., 2009; Szalay et al., 2012) as a control taste. In retrospect, incorporating water as a control solution has limited the functional contrasts that can be explored in the consummatory phase of the paradigm.

Whole brain statistical maps that corresponded to sucrose and stevia stimulus receipt events contrasted against those that corresponded to the “stimulus withheld” events were tested. Our statistical maps showed very nicely that receipt of both sucrose and stevia solution produced significant BOLD responses in oral somatosensory regions, the insula cortex as well as regions of the supplementary motor cortex and cerebellum VI area. Briefly, these statistical maps are in accordance with previous gustatory fMRI work and literature. The insula has been well documented in its role of gustatory perception and processing (Frank et al., 2013) within the brain and is commonly referred also as part of the primary gustatory cortex. From the insula, projections to the striatum, OFC and ACC carry the oral sensory information where properties regarding valence and other cognitive processes are encoded (Breslin, 2013). BOLD response increases were also seen within the cerebellum. Activity within the cerebellum has been observed from previous fMRI studies looking at taste (Small et al., 2003; Zald & Pardo, 2000). Small et al., reported cerebellar activity associated with intensity of substances and suggested a role of the cerebellum to respond to taste intensity information and guide oral movements (Small et al., 2003). Both taste responses displayed activation within the supplementary motor area (SMA). The SMA is well known for its role in controlling movement actions (Nachev et al., 2007) and therefore this response seen could be due to preparation for swallowing. The observation that these pre‐motor regions are active during the consummatory period can be explained as the receipt or delivery phase was contrasted against the delivery withheld period.

Sugar receipt or consummatory BOLD estimates extracted from the NAcc positively correlated with the receipt of sugar stimuli under insulin conditions, but unfortunately did not survive correction for multiple statistical tests. Aside from this relationship there were no reported differences between BMI groups or any associated effects of IN‐INS on sugar consummatory responses. Consummatory responses for stevia stimulus provided a differential effect of insulin across groups within a region that corresponded to parts of the ACC and the dorsal OFC, but unfortunately post hoc whole brain testing did not lead to any significant drug related changes within this cluster. We did however identify a group related response difference from ROI analysis of the amygdala for sugar consummatory response that existed only following IN‐INS delivery. In this case the data suggest that BOLD responsivity decreases for the normal weight group in comparison to overweight. The dorsal surface of the tongue is scattered with raised projections known as papillae which are lined with taste buds. Each taste bud hosts a large number of taste receptor cells with afferent axons that carry information along cranial nerves to the brainstem and thalamus where relay neurons connect with the insula cortex, amygdala and hypothalamus (Breslin, 2013). From preclinical and in vitro literature it is known that the central nucleus of the amygdala expresses an abundance of insulin receptors (Korol et al., 2018). Binding of insulin to these receptors has been shown to selectively increase both the amplitude and frequency of spontaneous inhibitory postsynaptic currents (Korol et al., 2018). The consequent reduction in oxygen demand in the amygdala leads to lower cerebral perfusion and may be what we see in our data.

This is the first time that a BOLD functional imaging protocol has been implemented to explore brain responsiveness to the receipt of the non‐nutritive sweetener, Stevia. The function of non‐nutritive, zero calorie, sweeteners as an alternative to energy dense sweeteners such as sugar for weight loss and promotion of health is an area of considerable debate (Mattes & Popkin, 2009). This work does not directly add knowledge to the literature regarding weight loss and health promotion but our data suggest that anticipation of tasting stevia and the actual taste response do not differ to that of sucrose. It is important to note that this study focused on the acute taste response and perhaps is not a full embodiment of consumption which have retro‐nasal elements (Landis et al., 2005). Anecdotal reports of the stevia taste from our volunteers said that it had a noticeable aftertaste that was more pronounced than sucrose. Anticipation of stevia produced a number of interesting insulin and BMI related effects that we have reported and commented on above. These observations were not mirrored for the sugar stimulus and we comment that this may be as a result of the novelty of such a taste stimulus and also the differences in aftertaste that may add to the differential effects seen.

To minimise potential habituation or order effects, we used several playlists for ordering and timing of stimuli and additionally implemented different fractal cue configurations across sessions. Furthermore, we also compared run 1 versus run 2 using a whole brain analysis and did not find any significant clusters. The authors would also like to comment on other methodological considerations. Despite observing significant group differences for some of the regions and for some of the contrasts; it is worth noting that we employed a relatively small number of subjects in this study. We may therefore lack sufficient statistical power to generalise our findings to a wider population. Some of the significant findings that we observed in the between‐subject comparisons may be more susceptible to the sample size employed; as the statistical power issue has a reduced impact in the within‐subjects comparisons.

## 5 CONCLUSION

This work is the first of its kind to explore the acute effects of increased central insulin on food cue reactivity paired with food stimuli receipt and should benefit future work which may explore these effects on patient groups with known impaired insulin signalling. In conclusion we have shown that age matched normal weight and overweight (not obese) individuals respond similarly to both anticipation and receipt of sugar and stevia solutions under placebo conditions. However, in the presence of increased central insulin concentrations, from intranasal delivery, marked differences to anticipation of sweet stimuli within the prefrontal cortex and basal forebrain regions are identified from these BMI differences as well as functional responsive differences seen in the amygdala when tasting stevia solution. As a post‐prandial hormone insulin reaches the brain several hours after food consumption. This work shines a light on regions involved in food evaluation and decision making that may be key to understanding how, from a cognitive perspective, an overweight BMI and phenotype may impact future weight gain and appetite and also to what extent increasing insulin may benefit cognitive and behavioural therapies for weight loss or management.

## AUTHOR CONTRIBUTIONS

Jed Wingrove—participant recruitment, study design, data collection, data analysis, manuscript production. Owen O'Daly—advised on fMRI study design, supported data analysis and editing of manuscript. Alfonso De Lara Rubio—main engineer for the pneumatic taste stimulus device. Simon Hill—main paradigm engineer for fMRI task. Magda Swedroska—provided pharmacy support and obtained reagent and insulin preparations. Ben Forbes—provided pharmacy support and obtained reagent and insulin preparations. Stephanie Amiel—supported overall study design and editing of manuscript. Fernando Zelaya—supported fMRI study design, fMRI analysis and editing of manuscript.

## FUNDING INFORMATION

EPSRC CASE EP/L015226/1 provided funding for the project and Jed Wingrove; financial support for the project was also provided by Unilever UK.

## CONFLICT OF INTEREST

The authors declare that this study received funding from Unilever UK. The funder was not involved in the study design, collection, analysis, interpretation of data, the writing of this article or the decision to submit it for publication.

## Supporting information


**Appendix S1** Supplementary Information

## Data Availability

The data that support the findings of this study are available from the corresponding author upon reasonable request.
