# Supplementary material for: The influence of insulin on anticipation and consummatory reward to food intake: A functional imaging study on healthy normal weight and overweight subjects employing intranasal insulin delivery
Source: Hum Brain Mapp. 2022 Jul 21;43(18):5432–51. doi: 10.1002/hbm.26019 (PMC9704782; doi:10.1002/hbm.26019)
Supplement: Supplementary file 1 — Appendix S1 Supplementary Information [file HBM-43-5432-s001.docx]

**Supplementary Material**

## Questionnaires

During screening visits two questionnaires were completed to assess eating behaviour and diet composition; the Three Factor Eating Questionnaire (TFEQ) and the Dietary Fat and Sugar (DFS) questionnaire, respectively. The TFEQ measures three eating behaviours or factors: dietary restraint, disinhibition and hunger (de Lauzon, Romon et al. 2004) and is a 36 part questionnaire that uses both true and false questions as well as 4-point and 5-point Likert scales. TFEQ scores for each factor were calculated for each participant. The DFS questionnaire provides an approximate guide to examine how often meals or food that contain high amounts of saturated fat and sugar are consumed. The DFS contains 26 food types for which the participant must mark on a scale how often that food type is consumed. The scale ranges from “less than once per month” to “5 + times per week” on a 5-point scale. The questionnaire is quick to complete and has been shown to produce valid and reliable measures of saturated fat and free sugar consumption when compared with weekly food diaries which are also more arduous on the participant (Francis and Stevenson 2013). Results for both these questionnaires were compared across groups using two sample t-tests and judged to *p* < 0.05 significance threshold.

|  | **Lean (n=10)** | **OW (n=14)** | ***P* value** |
| --- | --- | --- | --- |
| **Restraint** | 8.9 ± 5.3 | 9.6 ± 4.5 | 0.74 |
| **Inhibition** | 2.1 ± 0.6 | 5.9 ± 2.4 | 0.10 |
| **Hunger** | 4.8 ± 4.5 | 5.7 ± 3.2 | 0.57 |
| **Fat intake** | 55.2 ± 15.1 | 59.1 ± 10.3 | 0.45 |
| **Sugar Intake** | 10.4 ± 4.2 | 11.4 ± 3.2 | 0.53 |

Table S1. Demographics for the study cohort. Lean, normal weight. OW, overweight. BMI, body mass index. HOMA-IR, homeostatic model assessment-insulin resistance. *P* values have been calculated from two sample t-tests. *** significant *p* < 0.05. Data are presented as mean ± SD.

## Blood Analysis

Venous blood samples were spun in a centrifuge (10 mins at 1000 rpm). Plasma and serum were extracted into aliquots and stored at -20°C. Biochemical analysis was performed using routine assays to ascertain serum insulin and C-peptide (Siemens Healthcare Centaur Assays) and plasma glucose (Siemens Healthcare AVIDA) concentrations, respectively.

**Taste Delivery Hardware**

The taste delivery system is an automated, high speed, liquid delivery system that dispenses a calibrated bolus or taste stimulus of 0.5 mL. The dose is restricted to 0.5 mL to minimise any swallowing difficulties that may occur with a larger bolus whilst laying down. The system can be broken down into three constituent parts: 1) the Pneumatic control device which resides in the scanner control room, 2) the dispensing unit which houses the liquid reservoirs and calibrated dose syringes and 3) the delivery tube which extends from the main unit to the subject’s mouth. The mouthpiece has three outlets for the three tastes that were used during this task. With each taste being pumped from a separate reservoir and outlet, the contamination of tastes is largely avoided.

**Additional Discussion on TFEQ and dietary intake**

Eating behaviour and dietary intake of sugar and saturated fat, measured using TFEQ and DFS questionnaires did not provide any significant differences between the two groups but for all measures average scores were slightly greater in the OW group compared to lean. Previous research has found that BMI correlates positively with both TFEQ disinhibition and hunger factor scores, but not TFEQ dietary restraint (Zimmerman, Mason et al. 2017), however it must be noted that correlations between other methods of obtaining restraint and BMI do exist (Adams, Chambers et al. 2019). From previous research, Kullmann *et al.,* showed that disinhibition, measured through the TFEQ correlated with cerebral blood flow (CBF) responses to IN-INS within the prefrontal cortex (Kullmann, Heni et al. 2015). Correlations of TFEQ and dietary scores with functional measures were beyond the scope of this extensive work but would be useful to incorporate in future work.

**References**

Adams, R. C., C. D. Chambers and N. S. Lawrence (2019). "Do restrained eaters show increased BMI, food craving and disinhibited eating? A comparison of the Restraint Scale and the Restrained Eating scale of the Dutch Eating Behaviour Questionnaire." Royal Society open science **6**(6): 190174-190174.

de Lauzon, B., M. Romon, V. Deschamps, L. Lafay, J. M. Borys, J. Karlsson, P. Ducimetiere and M. A. Charles (2004). "The Three-Factor Eating Questionnaire-R18 is able to distinguish among different eating patterns in a general population." J Nutr **134**(9): 2372-2380.

Francis, H. and R. Stevenson (2013). "Validity and test–retest reliability of a short dietary questionnaire to assess intake of saturated fat and free sugars: a preliminary study." Journal of Human Nutrition and Dietetics **26**(3): 234-242.

Kullmann, S., M. Heni, R. Veit, K. Scheffler, J. Machann, H.-U. Häring, A. Fritsche and H. Preissl (2015). "Selective Insulin Resistance in Homeostatic and Cognitive Control Brain Areas in Overweight and Obese Adults." Diabetes Care **38**(6): 1044-1050.

Zimmerman, A. R., A. Mason, P. J. Rogers and J. M. Brunstrom (2017). "Obese and overweight individuals are less sensitive to information about meal times in portion-size judgements." International Journal Of Obesity **42**: 905.
